# Spatial–Temporal Characteristics, Source Apportionment, and Health Risks of Atmospheric Volatile Organic Compounds in China: A Comprehensive Review

**DOI:** 10.3390/toxics12110787

**Published:** 2024-10-29

**Authors:** Yangbing Wei, Xuexue Jing, Yaping Chen, Wenxin Sun, Yuzhe Zhang, Rencheng Zhu

**Affiliations:** 1 State Key Laboratory of Environmental Criteria and Risk Assessment, Chinese Research Academy of Environmental Sciences, Beijing 100012, China; wyb0110@gs.zzu.edu.cn; 2 Institute of Atmospheric Environment, Chinese Research Academy of Environmental Sciences, Beijing 100012, China; 3 School of Ecology and Environment, Zhengzhou University, Zhengzhou 450001, China; jingxuexue@gs.zzu.edu.cn (X.J.); cyptt@stu.zzu.edu.cn (Y.C.); sunwx123@gs.zzu.edu.cn (W.S.)

**Keywords:** VOCs, China, source apportionment, health risk assessment

## Abstract

Volatile organic compounds (VOCs) are ubiquitous in the atmosphere, posing significant adverse impacts on air quality and human health. However, current research on atmospheric VOCs mainly focuses on specific regions or industries, without comprehensive national-level analysis. In this study, a total of 99 articles on atmospheric VOCs in China published from 2015 to 2024 were screened, and data on their concentrations, source apportionment, and health risks were extracted and summarized. The results revealed that the annual average concentrations of TVOCs and their groups in China generally increased and then decreased between 2011 and 2022, peaking in 2018–2019. A distinct seasonal pattern was observed, with the highest concentrations occurring in winter, followed by autumn, spring, and summer. TVOC emissions were highly concentrated in northern and eastern China, mainly contributed by alkanes and alkenes. Source apportionment of VOCs indicated that vehicle sources (32.9% ± 14.3%), industrial emissions (18.0% ± 12.8%), and other combustion sources (13.0% ± 13.0%) were the primary sources of VOCs in China. There was a significant positive correlation (*p* < 0.05) between the annual mean VOC concentration and population size, and a notable negative correlation (*p* < 0.05) with GDP per capita. Atmospheric VOCs had no non-carcinogenic risk (HI = 0.5) but exhibited a probable carcinogenic risk (7.5 × 10^−5^), with relatively high values for 1,2-dibromoethane, 1,2-dichloroethane, and naphthalene. The health risk was predominantly driven by halocarbons. These findings are essential for a better understanding of atmospheric VOCs and for developing more targeted VOC control measures.

## 1. Introduction

As a major energy consumer and carbon contributor, China accounted for 18–35% of global air pollutant emissions between 2000 and 2014 [1]. To address the severe air pollution, the Chinese government has implemented stringent clean air policies in recent years, including legislating the National Air Pollution Prevention and Control Action Plan (2013), formulating the Three-Year Action Plan for Winning the Blue Sky War (2018–2020), and introducing the Air Quality Continuous Improvement Action Plan (2023). Hence, the emissions of SO_2_, NO_x_, CO, PM_2.5_, and NH_3_ decreased by 72.3%, 11.8%, 30.2%, 48.1%, and 3.2% in China from 2013 to 2020 [2]. However, photochemical pollutants, primarily dominated by O_3_, have shown an increasing trend, with the annual daily maximum 8 h average O_3_ concentrations increasing at a rate of 2.19  ppb yr^−1^ over 2014–2020 [3,4]. Furthermore, 23.3% of 337 prefecture-level cities still fail to meet the O_3_ standards according to the China Environmental Status Bulletin in 2023 [5]. O_3_ is produced by photochemical oxidation of volatile organic compounds (VOCs) catalyzed by hydroxyl radicals, resulting in a highly nonlinear relationship between atmospheric VOCs and O_3_ formation [6,7]. In most areas of China, including the Beijing–Tianjin–Hebei region, Guanzhong, and the Yangtze River Delta region, controlling atmospheric VOCs can effectively reduce the coordinated pollution of O_3_ and secondary organic aerosols [7,8,9].

VOCs are gaseous mixtures characterized by their high saturated vapor pressure and volatility at ambient temperatures. The predominant groups of VOCs include alkanes, alkenes, alkynes, aromatics, oxygenated VOCs (OVOCs), and halocarbons [10]. Atmospheric VOCs have been identified as significant contributors to the formation of photochemical smog, the promotion of environmental acidification, and the greenhouse effect [11,12]. Prolonged or recurrent exposure to VOCs may cause damage to the central nervous system, kidneys, and liver [13,14]. Moreover, certain VOCs, such as benzene, 1,3-butadiene, chloroethylene, and trichloroethylene, are classified as recognized carcinogens by the International Agency for Research on Cancer (IARC), possessing carcinogenic, genotoxic, mutagenic, and neurotoxic properties [15]. Hazardous VOCs, both carcinogenic and non-carcinogenic, account for 20–40% of all non-methane VOCs in China [16]. Thus, controlling atmospheric VOC emissions is critical for reducing atmospheric pollution and protecting human health in China.

Depending on the intensity of source emissions, topographic structures, and meteorological conditions, the distribution of VOC concentrations and their groups varies across regions in China. For example, atmospheric VOC concentrations measured in urban areas of Beijing in 2020 averaged 26.2 ± 16.4 ppbv, which were predominantly composed of alkanes (27.0%) [17]. Atmospheric VOCs in Jiaozuo, where a substantial amount of coal chemical industries exists, were slightly higher at 33.6 ± 6.4 ppbv than in Beijing, predominantly contributed by OVOCs, with a range of 22.5–40.2% [18]. VOCs are emitted from anthropogenic activities (vehicle sources, solvent usage, industrial processes, fuel and gas evaporation, and fossil fuel combustion) and biogenic sources (vegetation and wildfires) [19,20,21]. The sources of atmospheric VOCs vary among different cities, influenced by a variety of city sizes and industrial structures. Atmospheric VOCs in Shanghai primarily originated from vehicle sources (24.0–44.5%) and solvent use (10.4–31.4%) [22], while the main sources in Guiyang were fuel evaporation from stations and vehicles (23.4–42.7%) and household emissions (11.6–20.0%) [23]. Liquefied petroleum gas (LPG) fuel consumption was the largest contributor to atmospheric VOCs in Hong Kong, accounting for 60.0% ± 5%, since LPG was used as the primary clean fuel for most taxis and public and private minibuses [24]. Previous studies on atmospheric VOCs have primarily focused on specific regions or industries [25,26]. Although Li et al. [10] conducted a comprehensive review of anthropogenic VOC emissions in China, it emphasized emission inventory estimates rather than the online monitoring of VOCs. Therefore, it is essential to conduct a comprehensive nationwide analysis of atmospheric VOCs to address the limitations of previous studies, which will provide a broader perspective for future research.

In this study, we compared the emission characteristics of atmospheric VOCs in China through a systematic literature review, discussing their temporal trends and spatial distributions. The source apportionments reported in the literature were summarized and further analyzed by comparing the annual mean VOC concentrations with population size and per capita gross domestic product (GDP). The health risks posed by hazardous VOC components were evaluated. The findings of this study could enhance the scientific understanding of atmospheric VOC contamination levels and human health risks in China and provide some inspiration and recommendations on controlling and preventing VOC emissions effectively.

## 2. Materials and Methods

### 2.1. Eligibility Criteria and Search Method

**Figure 1:**
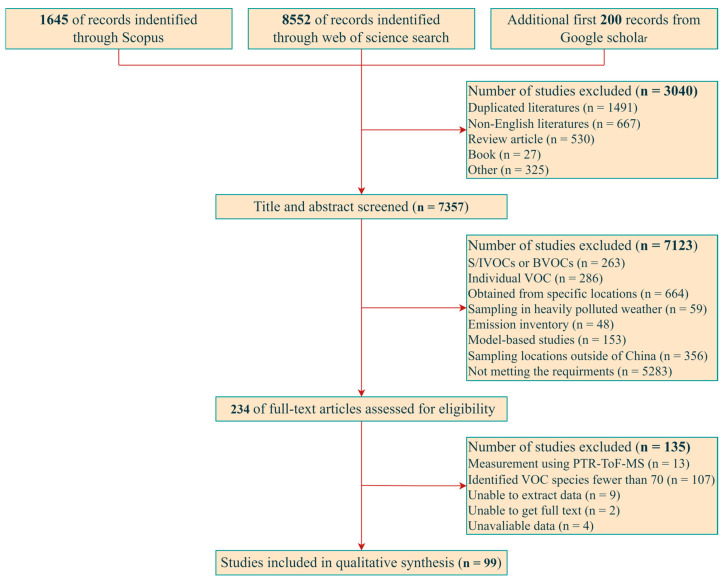
Flow diagram of study selection.

Peer-reviewed journal articles published from 1 January 2015 to 1 August 2024 to be examined were identified through a comprehensive search in Web of Science, Scopus, and Google Scholar. Literature on atmospheric VOCs in China was systematically reviewed using the following search terms: “atmospheric volatile organic compounds” or “atmospheric VOCs” and “China”. Two authors independently screened the titles and abstracts of 7357 records and the full texts of 99 published papers to ensure reliability and validity, as illustrated in Figure 1 [8,9,17,18,19,20,21,22,23,25,27,28,29,30,31,32,33,34,35,36,37,38,39,40,41,42,43,44,45,46,47,48,49,50,51,52,53,54,55,56,57,58,59,60,61,62,63,64,65,66,67,68,69,70,71,72,73,74,75,76,77,78,79,80,81,82,83,84,85,86,87,88,89,90,91,92,93,94,95,96,97,98,99,100,101,102,103,104,105,106,107,108,109,110,111,112,113,114,115].

To further identify articles that met the inclusion criteria of the present study, the following inclusion criteria were established: (i) full-text-available research articles with peer review and in English were included, while books, review articles, conference papers, and letters to editors were excluded; (ii) articles related to atmospheric VOCs; (iii) VOCs obtained in real-world environments. The exclusion criteria were the following: (i) VOCs measured from specific locations, such as airports, indoor environments, and industrial processes, as well as those tested only during high O3 or haze weather conditions; (ii) identified VOC species fewer than 70, or VOC groups fewer than 4; (iii) articles that did not include raw data; (iv) sampling locations outside of China; (v) model-based studies. A summary of the adopted studies is listed in Table S1. The methodological variability across studies inevitably leads to some degree of uncertainty. However, a rigorous literature selection process was employed in this study to minimize its potential impact.

### 2.2. Quality Assessment and Data Extraction

Literature quality assessment was conducted using criteria based on a meta-analysis [116]. Specifically, it was assessed by two researchers (Wei Y. and Jing X.) independently, and their disagreements were resolved by a third investigator (Zhu R.). Original data presented in the publications were directly extracted from tables, texts, and Supplementary Materials. For graphical data, the Digitizer in Origin was employed to extract information. If necessary, authors were contacted for additional information. From each included study, information on the authors, year of publication, sampling location, sampling duration, number of samples, the average concentration of total VOCs (TVOCs) and their groups, source apportionment, and carcinogenic and noncarcinogenic risk values was extracted and compiled. The concentrations of hazardous VOC species demonstrated in the literature were also extracted to assess health risks. Moreover, the units of VOC concentrations were further unified to ppbv, for an intuitive comparison across different atmospheric VOCs.

### 2.3. Data Analysis

The data in this study were obtained by calculating arithmetic averages after extraction from previous reports. To minimize errors caused by potential sample heterogeneity across the studies, the data were divided into different subgroups based on sampling years, seasons, regions, and provinces for analysis. Specifically, the sampling regions were categorized into six regions: north China, east China, central China, south China, southwest China, and northwest China, as listed in Table S2.

### 2.4. Health Risk Assessment

The cancerous and chronic non-cancerous health risks of human exposure to hazardous VOCs were evaluated by calculating the Inhalation Cancer Risk (*ICR*) and Hazard Quotient (*HQ*), which were proposed by United States Environmental Protection Agency [117]. The *ICR* is expressed by Equations (1) and (2), as the product of inhalation unit risk (*IUR*) and exposure concentration (*EC*):

(1) $$ICR=\sum {IUR}_{i}\times {EC}_{i}$$

(2) $${EC}_{i}=\frac{{CA}_{i}\times ET\times EF\times ED}{AT}$$

where ${IUR}_{i}$ denotes the inhalation unit risk, m^3^/μg; ${EC}_{i}$ is the adjusted exposure concentration of the species *i*, μg/m^3^; ${CA}_{i}$ is the concentration of the *i*th atmospheric VOCs, μg/m^3^; ET represents daily exposure time (3.7 h/d) [34]; EF is the annual exposure frequency (365 d/y); ED is the human lifespan exposure duration (74.8 y), which was obtained from the exposure factor handbook of the Chinese population (adult); and AT is the average time (74.8 × 365 × 24 h) [118]. The *ICR* is classified as “definite risk” (>1 × 10^−4^), “probable risk” (1 × 10^−5^–1 × 10^−4^), or “possible risk” (1 × 10^−6^–1 × 10^−5^).

*HI* is the sum of the hazard quotients (*HQ*) of the *i*th atmospheric VOCs, as follows:

(3) $$HI=\sum {HQ}_{i}$$

(4) $${HQ}_{i}=\frac{{EC}_{i}}{{RfC}_{i}}$$

where ${RfC}_{i}$ represents the acceptable exposure concentration of species *i*, μg/m^3^. Generally, adverse chronic non-cancerous effects are predicted to occur when the exposure concentration exceeds the acceptable concentration (*HQ* > 1). ${IUR}_{i}$ and ${RfC}_{i}$ are summarized in Table S3.

## 3. Results and Discussion

### 3.1. Characteristics of VOCs

#### 3.1.1. Temporal Characteristics

**Figure 2:**
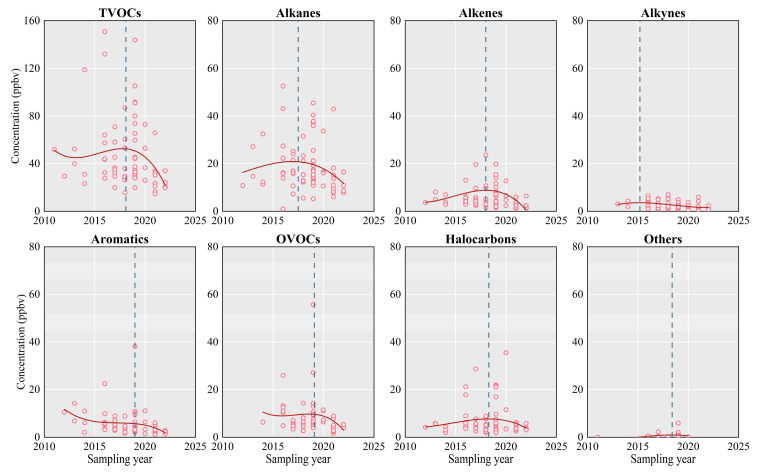
Time series of annual mean VOC concentrations from 2011 to 2022. The points, solid lines and dashed lines represent observation values, fitted curves, and the time of peak observation of VOCs, respectively.

The temporal variations in annual mean VOC concentrations in China from 2011 to 2022 are shown in Figure 2. The TVOCs and their groups generally follow an increasing then decreasing trend, peaking during 2018–2019. During this period, the annual mean concentration of TVOCs was 59.2 ± 32.8 ppbv, of alkanes was 21.6 ± 11.5 ppbv, of alkenes was 12.3 ± 25.3 ppbv, of alkynes was 2.2 ± 1.0 ppbv, of aromatics was 6.7 ± 8.0 ppbv, of OVOCs was 12.2 ± 12.5 ppbv, of halocarbons was 7.6 ± 6.9 ppbv, and of other compounds was 1.1 ± 1.7 ppbv. By 2022, the average annual concentration of TVOCs in China had decreased to 32.5 ± 6.2 ppbv, marking a 45.1% decrease, with the most dramatic declines observed in alkanes, alkenes, aromatics, and OVOCs, which dropped to 10.9 ± 3.9 ppbv (49.3%), 2.9 ± 2.4 ppbv (76.5%), 2.1 ± 0.6 ppbv (68.4%), and 4.9 ± 0.8 ppbv (59.8%), respectively. These results align with the VOC emission inventory reported by the European Union’s Community Emissions Data System, which showed a slightly decreasing trend during 2015–2019, with a reduction rate of 0.091 Tg yr^−1^ [10]. Wang et al. [119] also observed a consistent decline in anthropogenic VOC emissions in Chengdu between 2017 and 2020 (−5.9% yr^−1^). The more pronounced reduction in atmospheric VOC emissions observed in this study during the 2019–2022 period, compared to these emission inventories, can be attributed to the substantial suspension of industrial and other human activities during the COVID-19 pandemic. Furthermore, Simayi et al. [120] predicted that industrial VOC emissions may decrease by 5.0 Tg (30%) in 2030 compared to 2019 if all regions in China were to apply the same stringent control measures. Our findings reflect that the progressively stricter control measures in China have been effective in VOC emissions reduction.

In addition to anthropogenic activities, meteorological factors also influence the annual average concentrations of atmospheric VOCs. However, nationwide studies on the interannual variability of atmospheric VOCs driven by meteorological conditions in China remain limited, with most existing research focused on individual cities. Wang et al. reported that fine particulate matter (PM_2.5_) interannual variability associated with meteorological parameters in East Asia was 6.7%, primarily driven by changes in humidity, precipitation, and ventilation. Furthermore, due to extreme weather events caused by recent El Niño phenomena, the PM_2.5_ interannual variability associated with meteorological parameters in southern China reached as high as 12.0% [121]. Given that VOCs are one of the key precursors to PM_2.5_ [122], it is likely that VOCs are also influenced by similar meteorological processes, which affect their atmospheric reactions, transport, and removal. While changes in emissions remain the primary drivers of long-term VOC trends, the complex effects of meteorological conditions on atmospheric VOCs should be considered in future research.

**Figure 3:**
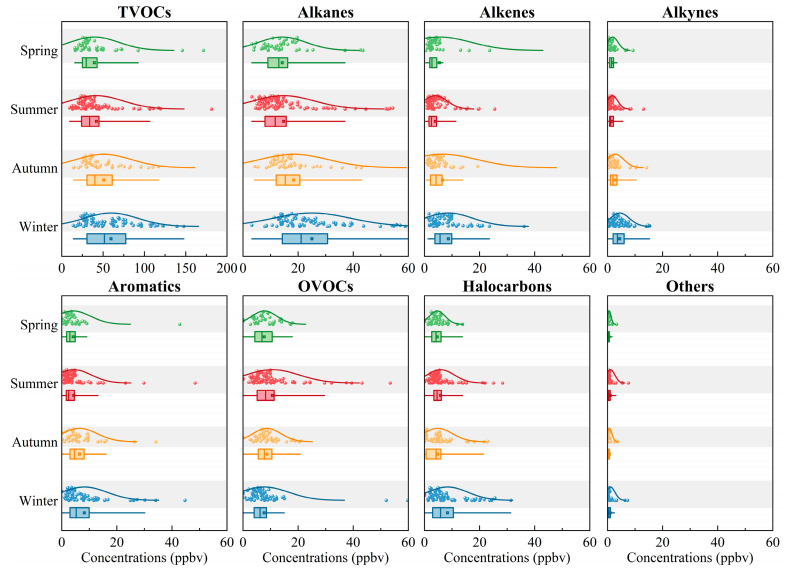
Seasonal variations in VOCs in China. The boxes represent the interquartile range, spanning from the 25th to 75th percentiles, the horizontal lines represent the median, and the dots in the box represent the mean value.

The seasonal variations in atmospheric TVOCs and their groups in China are plotted in Figure 3. In general, TVOC concentrations were estimated to be 39.6 ± 29.8 ppbv in spring, 41.9 ± 32.6 ppbv in summer, 50.8 ± 33.9 ppbv in autumn, and 59.6 ± 32.6 ppbv in winter, exhibiting a clear seasonal dependence with higher levels in winter and autumn and lower levels in spring and summer. The increased concentrations of TVOCs in autumn and winter are primarily caused by increased emissions from coal combustion for residential heating [23]. Furthermore, lower temperatures reduce the reactivity of TVOCs, leading to less removal through photochemical reactions and resulting in their persistence in the atmosphere [50]. The lower atmospheric mixing heights typical of winter also exacerbate this retention, contributing to the higher concentrations during these seasons [123]. This seasonal dependence was consistent with our findings on alkanes, alkenes, alkynes, aromatics, halocarbons, and other compounds, except that OVOCs reached the highest concentrations in summer. Most anthropogenic OVOCs are emitted directly by primary emission sources and formed through photochemical reactions [124]. This leads us to propose that high temperatures and low humidity in summer contributed a relatively high proportion of OVOCs by promoting the photochemical oxidation rates. Moreover, higher concentrations of OVOCs in the summer could be related to the intense primary industrial emissions, which are likely restricted during heavy pollution episodes in autumn and winter due to regulations limiting industrial activity [71]. For specific cities, there are certain differences in the seasonal distribution of VOCs. For instance, the peak TVOC concentration in Chongqing occurred in the summer of both 2014 and 2016, with isopentane, butane, benzene, and toluene contributing the most [38], while in Beijing, the peak TVOC concentration was observed in the autumn of 2019, with acetone, dichloromethane, n-butane, and toluene being the largest contributors [69]. It was speculated that the formation of VOCs is complex and easily influenced by daily temperature, relative humidity, planetary boundary layer height, terrain, topography, and other factors [23].

#### 3.1.2. Spatial Characteristics

**Figure 4:**
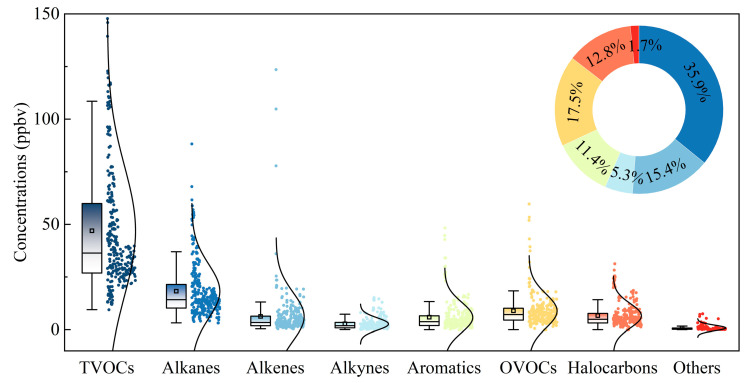
Distribution characteristics of TVOCs and their groups in China. The boxes represent the interquartile range, spanning from the 25th to 75th percentiles, the horizontal lines represent the median, and the dots in the box represent the mean value.

In general, alkanes (18.3 ± 12.6 ppbv) were the most abundant VOC group in China, comprising 35.9% of the TVOCs on average, followed by OVOCs (8.9 ± 8.0 ppbv; 17.5% of TVOC), alkenes (7.8 ± 18.0 ppbv; 15.4%), halocarbons (6.6 ± 6.0 ppbv; 12.8%), aromatics (5.8 ± 7.0 ppbv; 11.4%), alkynes (2.7 ± 2.9 ppbv; 5.3%), and other compounds (0.8 ± 1.3 ppbv; 1.7%), as shown in Figure 4.

**Figure 5:**
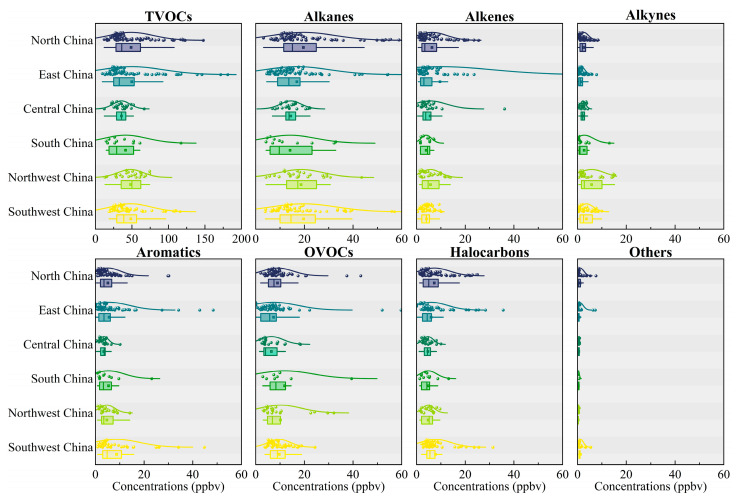
VOC concentrations across different regions in China. The boxes represent the interquartile range, spanning from the 25th to 75th percentiles, the horizontal lines represent the median, and the dots in the box represent the mean value.

Due to the discrepancies in economic development and population growth among the regions, the VOC emissions varied accordingly, as depicted in Figure 5. TVOC emissions were highly concentrated in north (50.9 ± 30.8 ppbv) and east China (49.9 ± 44.8 ppbv), mainly attributed to alkanes and alkenes, which was consistent with the VOC emission inventory established by Wu et al. [125] and Huo et al. [126]. Furthermore, the TVOC concentrations in northwest, south, southwest, and central China were 48.2 ± 17.4 ppbv, 41.3 ± 29.7 ppbv, 48.7 ± 27.5 ppbv, and 37.3 ± 11.6 ppbv, respectively. Note, however, that the measured VOC levels in southwest and northwest China may have been overestimated, as the reviewed literature did not include samples from the remote regions of Tibet and Xinjiang. Apart from alkanes, OVOCs accounted for the highest proportion of TVOCs in northwest (21.2%) and south China (28.9%). It was speculated that the high altitude and intense solar radiation in northwest China, along with the high temperature of south China, likely enhance the formation of OVOCs from primary emission sources through photochemical reactions [56,96]. In addition, Huang et al. reported that [23] relatively high OVOC emissions in Guizhou (southwest China), with α-pinene (0.98 ± 2.25 ppbv) notably exceeding the levels in other cities, may be influenced by rich forest resources and the local hilly terrain. Meanwhile, the greater contribution of aromatics to TVOCs in southwest China was dominated by vehicle emissions [89].

**Figure 6:**
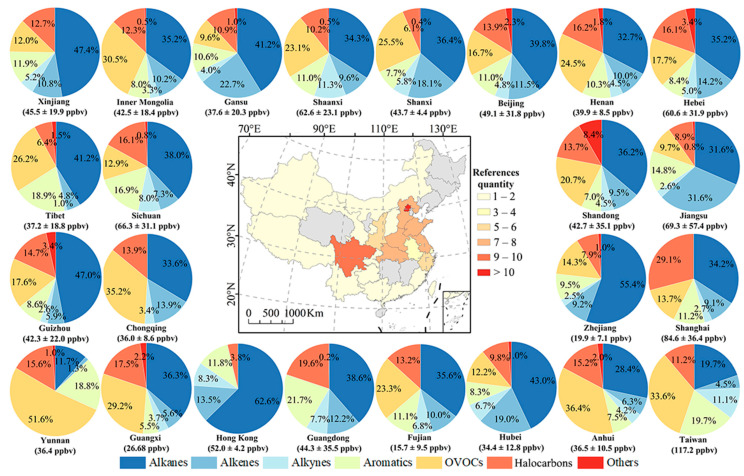
The frequencies of literature studies, the documented mean TVOC concentrations (mean ± std, ppbv), and the percentages of VOC groups in different provinces and municipalities in China.

Figure 6 reveals the frequencies of literature studies, the documented mean TVOC concentrations, and the percentages of VOC groups in different provinces and municipalities of China. This evidences that research on VOCs has primarily been concentrated in economically developed regions such as Beijing, Zhejiang, Jiangsu, and Sichuan, as well as the major transportation hubs like Hebei and Henan in recent years. The top five provinces or municipalities with the highest observed TVOC concentrations are as follows: Taiwan (117.2 ppbv), Shanghai (84.6 ± 36.4 ppbv), Jiangsu (69.3 ± 57.4 ppbv), Sichuan (66.3 ± 31.1 ppbv), and Shaanxi (62.6 ± 23.1 ppbv). The differences in VOCs among those provinces and municipalities were influenced by emission sources, sampling locations, population density, local regulation, and meteorological conditions, among other factors. For example, the high abundance of VOCs in Shanghai and Jiangsu was directly influenced by the large number of vehicles and high population density [41,127]. Sichuan is a lowland region surrounded by different-scale topography of plateaus and mountains, which block the downward transport of momentum in the upper troposphere, resulting in weak mechanical turbulence that inhibits the outward diffusion of VOCs [128]. Higher concentrations of isoprene and α-pinene were detected in Sichuan’s cities such as Dazhou and Chengdu, suggesting that they might stem from natural emissions [89]. In addition, in the major cities of Shaanxi Province, Xi’an observed higher proportions of formaldehyde and glyoxal, while Xianyang showed elevated acetone levels, and Hancheng emitted more cycloalkanes and isoprene. These typical industrial-source VOCs demonstrate that industrial activities have a significant influence on VOC emissions in Shaanxi [8,66].

**Figure 7:**
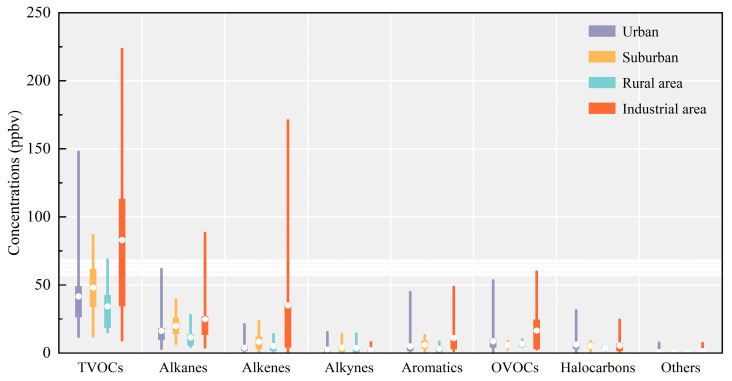
Contribution of TVOCs and their groups in urban, suburban, rural, and industrial areas. The boxes represent the interquartile range, spanning from the 25th to 75th percentiles, the horizontal lines represent the median, and the dots in the box represent the mean value.

Considering that VOC emissions were influenced by the functionality of different regions, the concentrations of TVOCs and their groups in various functional districts were summarized, as shown in Figure 7, revealing that TVOCs (82.0 ± 58.7 ppbv) were highest in industrial areas, followed by suburban (48.1 ± 20.6 ppbv), urban (41.9 ± 24.8 ppbv), and rural areas (34.3 ± 21.8 ppbv). Interestingly, although suburban TVOC emissions appeared to be lower than those in urban areas, urban OVOC emissions were observed to be slightly higher than those in the suburbs. It is hypothesized that this phenomenon may be influenced by the urban heat island effect. Firstly, the warm temperatures associated with the urban heat island in urban areas enhance turbulent mixing and increase the height of the urban boundary layer, potentially facilitating the upward dispersion of VOCs and leading to lower near-surface concentrations of VOCs [129]. Secondly, although the heat dome formed by the urban heat island may restrict the dispersion of pollutants, the increased temperatures are likely to accelerate the reaction of VOCs with hydroxyl radicals, leading to the formation of ozone and OVOCs, particularly from alkenes and aromatics [130]. Given the limited studies currently conducted in suburban areas, which are mostly concentrated in industrialized provinces such as Shanxi [66], Hebei [83], and Shandong [42], a broader range of samples is needed in future studies to substantiate the inference.

### 3.2. Source Apportionment of VOCs

#### 3.2.1. Variations in Source Emissions

**Figure 8:**
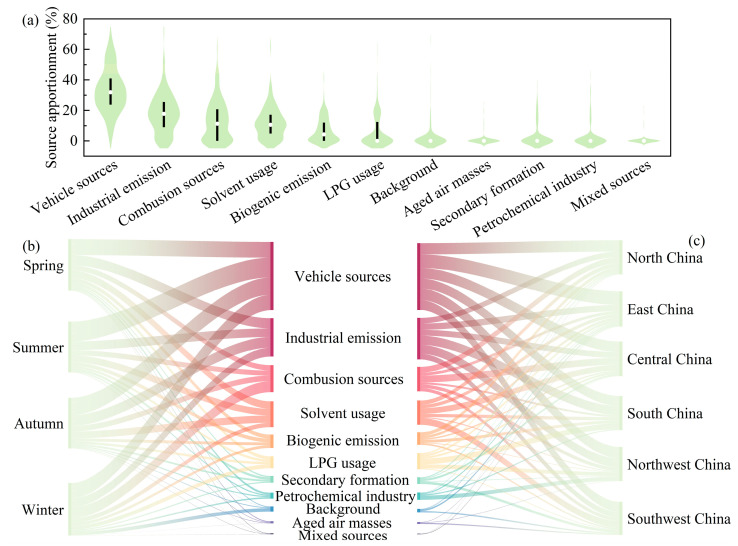
Source analyses of VOCs identified using the PMF model from various studies. (**a**) Violin plot of the main sources of VOCs in China. Wider areas in the violin plot represent an increased frequency at that y-value; the dots represent the mean value; and the thick vertical lines indicate the 75% confidence interval. (**b**,**c**) The intensity of VOC sources in different seasons and regions.

To effectively control VOC emissions, it is crucial to identify their sources. In the current research, there are four main methods to identify VOC sources, including positive matrix factorization (PMF), ratio analysis, principal component analysis, and chemical mass balance [69,131,132]. Among the above models, PMF has been extensively employed in recent VOC source apportionment studies in China. Figure 8a presents the common sources of atmospheric VOCs in China with the following: vehicle sources (32.9% ± 14.3%), industrial emissions (18.0% ± 12.8%), combustion sources (13.0% ± 13.0%), solvent usage (11.8% ± 10.0%), biogenic emission (6.8% ± 7.9%), LPG usage (5.9% ± 10.2%), secondary formation (3.3% ± 7.5%), petrochemical industry (2.7% ± 7.7%), background (2.2% ± 8.2%), aged air masses (0.8% ± 3.6%), and mixed sources (0.5% ± 2.8%). Moreover, there are some specific sources of VOCs. For instance, Huang et al. [133] suggested that pesticides (0.9–21.6%) are one of the contributing sources of atmospheric VOCs in Ningbo’s urban area, due to the high concentration of m-dichlorobenzene. Liu et al. [77] found that atmospheric VOCs measured in Beijing were enriched with n-undecane and species with long-chain hydrocarbons, attributed to asphalt emissions (6.9%), which may be related to the intensive infrastructural construction, such as road and airport construction, carried out in southern Beijing.

There are some differences in relative contributions from various VOC sources across different seasons (Figure 8b). Combustion sources contributed considerably more atmospheric VOCs in winter (21.0% ± 14.3%) than in summer (9.8% ± 13.4%), driven by the increased heating demand in winter [97]. Interestingly, some previous studies have divided motor vehicle sources into exhaust emissions and fuel evaporation [44,82]. Fuel evaporation contributed slightly more in summer (7.3% ± 10.3%) than in winter (2.1% ± 6.5%) due to the higher saturated vapor pressure of gasoline at elevated temperatures [134].

Apart from vehicle sources being the largest contributor, the sources of VOCs vary across different regions in China (Figure 8c). Notably, VOC emissions from combustion sources are highest in northern China, reaching 16.9% ± 14.4%. Industrial emissions and solvent usage contributed 22.1% ± 9.7% and 16.7% ± 8.7% of atmospheric VOCs in central China, respectively, which was associated with substantial numbers of local industrial enterprises, such as paint factories, rubber plants, plastic manufacturers, machinery factories, and printing facilities [18,60]. Meanwhile, northwest China was heavily influenced by the petrochemical industry [98].

#### 3.2.2. The Relationship of VOCs with the Population and per Capita GDP

**Figure 9:**
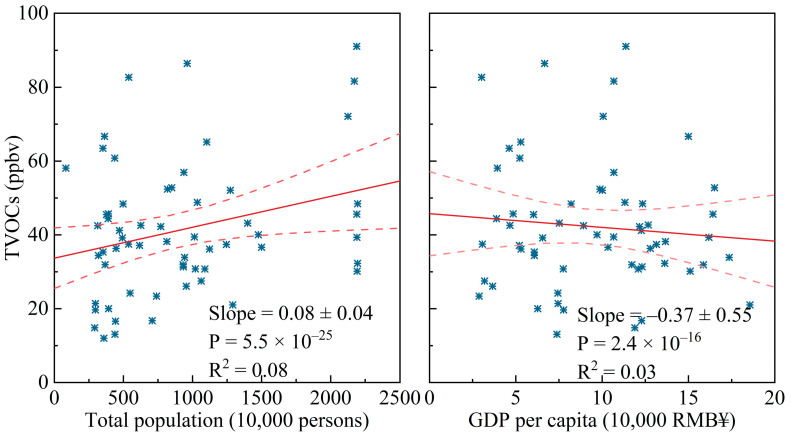
The relationship between the annual average atmospheric TVOC concentrations reported from 41 cities in China and the urban population as well as per capita GDP during the sampling year.

Given the numerous anthropogenic factors such as vehicle sources and industrial emissions bounded by population size and GDP per capita, the relationship between the population and GDP per capita of the sampled cities and the annual average TVOC concentrations is evaluated in this section, as shown in Figure 9. The urban population and per capita GDP data for the sampling years in Table S4 were obtained from the official websites of the respective provincial statistical bureaus of statistics. There was a positive correlation (*p* < 0.05) between the measured annual mean VOC concentration and population size, while a negative correlation (*p* < 0.05) was found between the annual mean VOC concentration and GDP per capita in these cities, which align with the trends in population- and GDP-weighted emissions previously identified by Li et al. [10]. While the associations were statistically significant, the relatively low R^2^ values suggest that the strength of these relationships may be limited by other unaccounted factors. It could be inferred that frequent human activities contribute to the generation of VOCs. In addition, cities with a higher per capita GDP tend to invest more in pollution control, effectively reducing VOC emissions through improved technical control measures, such as new energy vehicles and novel industrial VOC reduction technologies [135]. Hence, controlling VOC emissions should be prioritized in cities with large populations but a relatively low per capita GDP.

### 3.3. Health Risk

**Figure 10:**
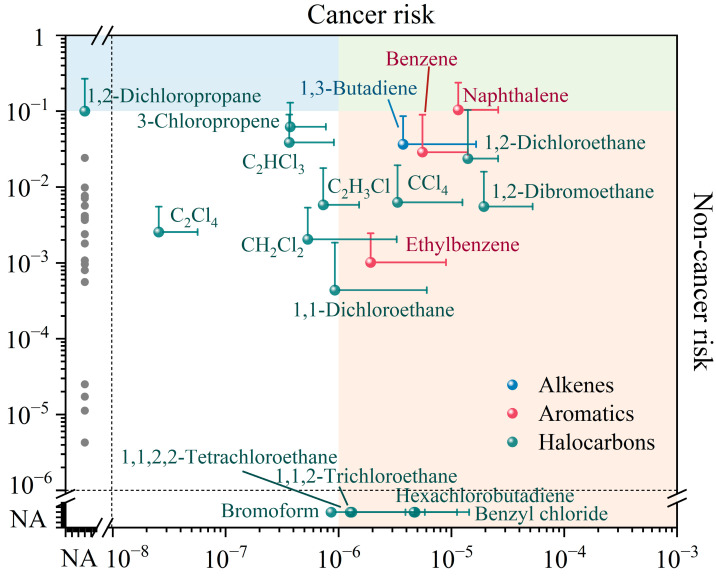
Inhalation cancerous risk and non-cancerous risk of atmospheric hazardous VOCs in China. Grey dotted lines indicated areas applicable exclusively to cancerous or non-cancerous risk. The blue or pink areas highlight VOC species with only potential non-cancerous or cancerous risks, while the green areas emphasize those VOC species exhibiting both risks. Grey dots represent VOC species without cancerous and non-cancerous risks. NA represent not available.

The potential risk of chronic diseases or cancers as a consequence of exposure to 39 hazardous VOCs in outdoor environments was evaluated using HI and ICR values, as demonstrated in Figure 10. The concentrations and carcinogenic and non-carcinogenic risks for the hazardous VOC group are plotted in Figure S1. This indicates that exposure to outdoor atmospheric VOCs does not pose a non-cancerous risk, for instance, with the results of a HI less than 1 (HI = 0.5). Among the hazardous VOCs, alkanes exhibited the highest content (36.5%), yet demonstrated a relatively low non-cancerous risk, contributing only 2.2%. In contrast, halocarbons (56.7%) and aromatics (28.4%) constituted the primary contributors to the HI, with concentration proportions of 33.5% and 28.0%, respectively. For individual species, the HQ values of all components were less than 1. Nevertheless, a HQ greater than 0.1 indicated that the components may pose a potential health risk, according to Zeng et al. [136]. Therefore, naphthalene (HQ = 0.2) and 1,2-dichloropropane (HQ = 0.1) should not be ignored. In addition, a few VOC species detected in certain regions of China exceeded the safety threshold. The non-carcinogenic risk of acrolein in Beijing reached as high as 25.5 in the autumn of 2019 [33]. Five VOC species were observed to potentially pose a non-cancerous risk to the residents in an industrial area situated in Shandong, namely propanal (5.12), 1,3-butadiene (2.04), n-heptane (1.66), 1,2-dichloropropane (1.16), and naphthalene (1.11) [48]. Therefore, the non-health risks of VOCs should not be ignored.

Regarding cancerous risk, it was striking that the total ICR value of VOCs observed in this study was 7.5 × 10^−5^, indicating a probable carcinogenic hazard for adults exposed to outdoor air. Among the VOCs, halocarbons and aromatics posed probable cancerous risks, while alkenes presented possible cancerous risks, with ICR values at 7.5 × 10^−5^ (69.8%), 5.3 × 10^−5^ (25.2%), and 3.7 × 10^−6^ (5.0%), respectively (Figure S1). For individual species, the carcinogenic risk of more than half of the total species exceeded the possible risk level of 1.0 × 10^−6^ but remained below the tolerable risk threshold of 1.0 × 10^−4^. Specifically, 1,2-dibromoethane (1.9 × 10^−5^), 1,2-dichloroethane (1.4 × 10^−5^), and naphthalene (1.1 × 10^−5^) emerged as the primary carcinogens, while benzene (5.5 × 10^−6^), benzyl chloride (4.8 × 10^−6^), hexachlorobutadiene (4.7 × 10^−6^), 1,3-butadiene (3.7 × 10^−6^), carbon tetrachloride (3.3 × 10^−6^), ethylbenzene (1.9 × 10^−6^), 1,1,2,2-tetrachloroethane (1.3 × 10^−6^), and 1,1,2-Trichloroethane (1.3 × 10^−6^) should not be overlooked. Notably, reports on the cancerous risks of atmospheric VOCs in China remain limited, primarily focusing on regions with high population densities and frequent industrial and commercial activities, such as Beijing [33], Shanghai [50], and the Yangtze River Delta [20]. The ICR values in this study were high, and the carcinogenic risk should be investigated thoroughly in the future combined with the VOC emissions from regions with fewer human activities.

The non-cancerous and cancerous risks of different regions and seasons in China in previous reports were also statistically investigated in this study (Figures S2 and S3). The non-cancerous risk was relatively higher in central China, while the cancerous risk was comparatively higher in southern China. Both non-cancerous and cancerous risks of most VOCs tended to increase as the ambient temperature decreased, correlating with higher VOC emission intensities during autumn and winter. Nevertheless, the health risk may decrease under real ambient conditions, given the shorter duration of inhalation exposure and the decrease in the frequency of outdoor activities [33]. The ICR values of some halocarbons, including 1,2-dibromoethane (1.8 × 10^−5^) and 1,2-dichloroethane (3.8 × 10^−5^), exhibited a more pronounced decrease. Although the use of these compounds in agriculture has been strictly regulated, residual or legacy deposits of these fumigants may still be present in soils. The higher summer temperatures likely lead to increased volatilization of these soil-bound residues [137], contributing to the more significant deterioration in their ICR values observed during this period. Hence, targeted control measures should be implemented for different regions and seasons to prevent the VOC health risk.

## 4. Conclusions

In this study, a total of 99 studies on atmospheric VOCs in China reported from 2015 to 2024 were screened, and the data on their concentrations, source resolutions, and health risks were extracted and summarized. The results demonstrated that the annual average concentrations of TVOCs and their groups in China between 2011 and 2022 essentially followed an increasing and then decreasing trend, peaking in 2018–2019. Meanwhile, TVOCs showed a clear seasonal dependence as follows: winter > autumn > spring > summer, which was consistent with their groups, except for OVOCs. TVOC emissions were highly concentrated in north and east China, mainly contributed by alkanes and alkenes. The top five provinces or municipalities with the highest observed TVOC concentrations are Taiwan, Shanghai, Jiangsu, Sichuan, and Shanxi, suggesting that VOCs in these regions should be prioritized for focused prevention and control.

The source apportionment using PMF from previous reports was analyzed. Among the sources, we found that vehicle sources, industrial emissions, combustion sources, and solvent use were the main contributors of VOCs in China. Combustion sources had a greater impact in winter, while fuel evaporation played a larger role in summer. There was a significant positive correlation between the measured annual mean VOC concentration and population size, while a remarkable negative correlation was observed between the annual mean VOC concentration and GDP per capita.

The health risk of VOCs was primarily influenced by halocarbons. None of the VOC species involved in the health risk assessment posed non-carcinogenic risks, but over half of the species exhibited probable carcinogenic risks, with 1,2-dibromoethane, 1,2-dichloroethane, and naphthalene displaying relatively high ICR values. The findings of this study could have significance for enhancing the understanding of atmospheric VOCs and controlling their emissions. Based on this research, future studies should pay more attention to assessing the effects of VOCs on human health.

## Data Availability

The original contributions presented in this study are included in the article/Supplementary Materials, and further inquiries can be directed to the corresponding authors.

## Supplementary Materials

The following supporting information can be downloaded at https://www.mdpi.com/article/10.3390/toxics12110787/s1, Table S1: List of 99 valid articles in this study; Table S2: Classification of sampling regions; Table S3: Summary of inhalation risks of hazardous VOCs; Table S4: The urban population and per capita GDP data for the sampling years; Figure S1: Concentrations (left), non-cancerous risks (middle), and cancerous risks (right) of different atmospheric hazardous VOC groups in China; Figure S2: The non-cancerous risks of different regions and seasons in China (N.D. represents not detected); Figure S3: The cancerous risks of different regions and seasons in China (N.D. represents not detected).
