# Supplementary material for: Spatial–Temporal Characteristics, Source Apportionment, and Health Risks of Atmospheric Volatile Organic Compounds in China: A Comprehensive Review
Source: Toxics. 2024 Oct 29;12(11):787. doi: 10.3390/toxics12110787 (PMC11598060; doi:10.3390/toxics12110787)
Supplement: Supplementary file 1 [file toxics-12-00787-s001.zip › toxics-3240025-supplementary.pdf]

Supplementary Materials

Spatial-temporal characteristics, source apportionment and health risks of atmospheric volatile organic compounds in China: A comprehensive review

Yangbing Wei [1,2,3], Xuexue Jing [3], Yaping Chen [3], Wenxin Sun [3], Yuzhe Zhang [1,2,*], Rencheng Zhu [3,*]

[1] State Key Laboratory of Environmental Criteria and Risk Assessment, Chinese Research Academy of Envi-ronmental Sciences, Beijing 100012, China

[2] Institute of Atmospheric Environment, Chinese Research Academy of Environmental Sciences, Beijing 100012, China

[3] School of Ecology and Environment, Zhengzhou University, Zhengzhou 450001, China

*Correspondence: Yuzhe Zhang (zhang.yuzhe@craes.org.cn) and Rencheng Zhu (zhurc@zzu.edu.cn)

Number of Pages: 22

Number of Supplementary Tables: 4

Number of Supplementary Figures: 5

Table S1. List of 99 valid articles in this study.

NO.	First authors	Years	Sampling duration	Sampling locations	Number		References
					VOCs groups	VOCs species	
1	Zhang et al.	2025	July to October 2020	Hebei	6	115	[1]
2	Wang et al.	2024	January to December 2019	Zhejiang	5	78	[2]
3	Wang et al.	2024	June 2021	Shandong	6	-	[3]
4	Hao et al.	2024	June and December 2021	Shandong	6	96	[4]
5	Ren et al.	2024	April to October 2020	Shanghai	6	106	[5]
6	Zhang et al.	2024	December 2013 to January 2014 and December 2021 to January 2022	Jiangsu	5	79	[6]
7	Lyu et al.	2024	January 2021 to August 2022	Zhejiang	7	98	[7]
8	Yan et al.	2024	August 2020	Tibet	5	146	[8]
9	Zuo et al.	2024	summer, autumn and winter of 2019-2020	Beijing	6	100	[9]
10	Liao et al.	2024	spring and autumn in 2019	Fujian	6	-	[10]
11	Li et al.	2024	May to October 2021	Henan	6	116	[11]
12	Liu et al.	2024	January, April, July, and October of 2016-2020	Beijing	7	89	[12]
13	Xie et al.	2023	Spring 2021	Yunnan	7	100+	[13]
14	Liu et al.	2023	May to June 2021	Beijing	5	95	[14]
15	Yu et al.	2023	August 2020	Anhui	7	116	[15]
16	Luo et al.	2023	2012 to 2017	Sichuan	5	82	[16]
17	Wang et al.	2023	November to December 2021	Shandong	7	106	[17]
18	Wang et al.	2023	June 2021 and September 2021	Anhui	6	80	[18]
19	Liu et al.	2023	August to October 2018	Jiangsu	7	99	[19]
20	Zhang et al.	2023	October to December 2018	Henan	6	102	[20]
21	Xu et al.	2023	January 2021	Hubei	6	106	[21]
22	Han et al.	2023	July to August 2019	Beijing	6	79	[22]
23	Wang et al.	2023	June to August 2019	Sichuan	6	122	[23]
24	Zhang et al.	2023	April to October 2022	Jiangsu	6	115	[24]
25	Guan et al.	2023	January to November 2020	Hebei	5	85	[25]
26	Zheng et al.	2023	June to August 2020	Zhejiang	5	98	[26]
27	Zhang et al.	2023	December 2021 to October 2022	Shandong	6	111	[27]
28	Wang et al.	2023	December 2020	Shandong	7	77	[28]
29	Ye et al.	2023	May to June 2021	Tibet	7	98	[29]
30	Wang et al.	2023	August, October and December in 2019	Beijing, Hebei and Shanghai	6	99	[30]
31	Zeng et al.	2023	July to September 2021	Henan	7	115	[31]
32	Li et al.	2023	January to February 2021	Zhejiang	6	116	[32]
33	Wu et al.	2023	July to August 2016	Beijing	6	99	[33]
34	Zhang et al.	2023	February 2021	Hubei	5	90	[34]
35	Xu et al.	2023	2017 to 2019	Zhejiang	6	98	[35]
36	Yang et al.	2023	September 2021	Gansu	7	115	[36]

37	Liu et al.	2023	August 2019	Beijing	6	-	[37]
38	Kong et al.	2022	January 2021	Sichuan	5	112	[38]
39	Liu et al.	2022	2019 to 2020	Shanxi	7	115	[39]
40	Huang et al.	2022	December 2018, January, April, July, October and November 2019	Guizhou	7	113	[40]
41	Huang et al.	2022	August to September 2020	Henan	6	106	[41]
42	Xue et al.	2022	June to September 2018	Shanxi	5	77	[42]
43	Yao et al.	2022	April to July 2020	Henan	6	74	[43]
44	Liu et al.	2022	August to September 2019	Shandong	7	122	[44]
45	Zhou et al.	2022	November 2020	Beijing	7	116	[45]
46	Chen et al.	2022	February to March 2020	Shanxi	5	77	[46]
46	Li et al.	2022	June to July 2021	Henan	7	115	[47]
48	Li et al.	2022	September 2017 to January 2018	Shanxi	7	102	[48]
49	Guo et al.	2022	June to July 2019	Gansu	7	87	[49]
50	Li et al.	2022	January to December 2018	Beijing	7	99	[50]
51	Cui et al.	2022	December 2018 to November 2019	Beijing	5	95	[51]
52	Manager et al.	2022	April and September in 2018	Fujian	6	100	[52]
53	Li et al.	2022	September and November to December 2017	Shanxi	6	102	[53]
54	Wang et al.	2022	July to December 2019	Anhui	7	117	[54]
55	Li et al.	2021	May to June 2018	Hubei	6	82	[55]
56	Yao et al.	2021	April to May 2019	Beijing	6	101	[56]
57	Wang et al.	2021	May to September 2019	Hebei	5	83	[57]
58	Xiong et al.	2021	June 2018 and January 2019	Sichuan	5	70	[58]
59	Liu et al.	2021	January, April, July, and October in 2016	Beijing	7	99	[59]
60	Yao et al.	2021	May to June 2019	Hebei	5	78	[60]
61	Xie et al.	2021	October to April 2018	Inner Mongolia	7	116	[61]
62	Xie et al.	2021	November 2018 to March 2019	Hebei	6	94	[62]
63	Wang et al.	2021	December and February in 2019, and April to May 2020	Jiangsu	5	102	[63]
64	Mozaffar et al.	2021	2018 to 2020	Jiangsu	7	100	[64]
65	Yang et al.	2021	2017 to 2018	Beijing, Hebei and Shandong	6	99	[65]
66	Song et al.	2021	June to July 2019	Shanxi	7	99	[66]
67	Zhang et al.	2021	July 2016	Beijing	7	99	[67]
68	Zhang et al.	2021	December, 2019	Henan	7	106	[68]
69	Jia et al.	2021	September 2018 to August 2019	Jiangsu	6	-	[69]
70	Mozaffar et al.	2020	July 2018	Jiangsu	6	89	[70]
71	Wang et al.	2020	January 2018	Sichuan	5	109	[71]
72	Tan et al.	2020	May 2016 to January 2017	Sichuan	7	99	[72]

73	Guan et al.	2020	April to August 2018	Hebei	7	117	[73]
74	Li et al.	2020	December 2015 to January 2016 and July to August 2016	Beijing	7	99	[74]
75	Hui et al.	2020	April to June 2017	Hubei	7	102	[75]
76	Liu et al.	2020	January, April, July, and October in 2016	Beijing	7	99	[76]
77	Tsai et al.	2020	July 2014	Taiwan	6	87	[77]
78	Fu et al.	2020	August to November 2018	Guangxi	7	107	[78]
79	Simayi et al.	2020	May 2016 to January 2017	Sichuan	6	-	[79]
80	Liang et al.	2020	January to December 2017	Tibet	6	102	[80]
81	Zhang et al.	2020	November 2017 to January 2018	Hebei	6	96	[81]
82	Li et al.	2020	May 2018	Henan	6	103	[82]
83	Sun et al.	2019	July 2018	Shanxi	6	86	[83]
84	Song et al.	2019	October to November 2014	Guangdong	7	103	[84]
85	Deng et al.	2019	August to October 2016	Sichuan	7	94	[85]
86	Liu et al.	2019	May 2017	Shanghai	5	108	[86]
87	Yang et al.	2019	September 2016 to August 2017	Hubei	5	100	[87]
88	Widiana et al.	2019	August 2010 to June 2011	Taiwan	5	-	[88]
89	Ge et al.	2019	December 2015 to January 2016 and July to 25 August 2016	Beijing	7	99	[89]
90	Yang et al.	2019	November 2017 to January 2018	Hebei	7	99	[90]
91	Han et al.	2018	November 2015 to June 2016	Jiangsu	5	-	[91]
92	Gao et al.	2018	February to March 2013	Beijing	6	90	[92]
93	Hui et al.	2018	September 2016 to August 2017	Hubei	7	102	[93]
94	Li et al.	2018	August to September 2015	Sichuan	7	96	[94]
95	Zhang et al.	2017	April 2014 to January 2015	Beijing	5	84	[95]
96	Wu et al.	2016	October 2014	Beijing	7	108	[96]
97	Lyu et al.	2016	February 2013 to October 2014	Hubei	6	99	[97]
98	Li et al.	2016	August to September 2015	Beijing	7	95	[98]
99	Li et al.	2015	October to November 2014	Beijing	6	102	[99]

Table S2 Classification of sampling regions.

Regions	Provinces and municipalities
north China	Beijing, Hebei, Heilongjiang, Jilin, Liaoning, Inner Mongolia, Tianjin, Shanxi, Shandong,
east China	Anhui, Jiangsu, Jiangxi, Zhejiang
central China	Henan, Hubei, Hunan
south China	Fujian, Guangxi, Guangdong, Hainan, Hong Kong, Macau, Taiwan
southwest China	Gansu, Ningxia, Qinghai, Xinjiang, Shaanxi
northwest China	Chongqing, Guizhou, Tibet, Sichuan, Yunnan

Table S3. Summary of inhalation risks of hazardous VOCs.

VOC species	CAS	IUR_i (m^3/μg)	RfC_i (μg/m^3)
n-Pentane	109-66-0		1.00E+03
n-Hexane	110-54-3		7.00E+02
n-Heptane	142-82-5		4.00E+02
n-Nonane	111-84-2		2.00E+01
Cyclohexane	110-82-7		6.00E+03
Methylcyclohexane	108-87-2		3.00E+03
Propene	115-07-1		3.00E+03
1,3-Butadiene	106-99-0	3.00E-05	2.00E+00
1,1,1-Trichloroethane	71-55-6		5.00E+03
1,1,2-Trichloroethane	79-00-5	1.60E-05	
1,1,2,2-tetrachloroethane	79-34-5	7.4E-6	
1,1-Dichloroethane	75-34-3	1.60E-06	5.00E+02
Dichloromethane	75-09-2	1.00E-8	6.00E+02
1,2,4-Trichlorobenzene	120-82-1		2.00E+02
1,2-Dibromoethane	106-93-4	6.00E-04	9.00E+00
1,2-Dichloroethane	107-06-2	2.60E-05	2.40E+03
1,2-Dichloropropane	78-87-5		4.00E+00
3-Chloropropene	107-05-1	6.00E-06	1.00E+00
Benzene	71-43-2	7.80E-06	3.00E+01
Benzyl chloride	100-44-7	4.90E-05	
Bromoform	75-25-2	1.10E-06	
Bromomethane	74-83-9		5.00E+00
Carbon tetrachloride	56-23-5	6.00E-06	1.00E+02
Chlorobenzene	108-90-7		1.00E+03
Chloroethane	75-00-3		1.00E+04
Chloroethene	1975-1-4	8.80E-06	1.00E+02
Chloroform	67-66-3		9.80E+01
Chloromethane	74-87-3		9.00E+01
Ethylbenzene	100-41-4	2.50E-06	2.60E+02
Hexachlorobutadiene	87-68-3	2.20E-05	
iso-Propylbenzene	98-82-8		4.00E+02
Naphthalene	91-20-3	3.40E-05	3.00E+00
p-Dichlorobenzene	106-46-7	1.10E-05	6.00E+01
Styrene	100-42-5		1.00E+03
Tetrachloroethene	127-18-4	2.60E-07	4.00E+01
Toluene	108-88-3		5.00E+03
Trichloroethene	1979-1-6	4.10E-06	2.00E+00
Xylenes	1330-20-7		1.00E+02
Methyl tert-butyl ether	1634-04-4		5.00E+00

Table S4. The urban population and per capita GDP data for the sampling years.

Sample city	Sample year	urban population (10,000 persons)	per capita GDP (10,000 RMB¥)
Baoding	2019	1063.0	31856
Beijing	2020	2189.0	164158
	2019	2190.1	161776
	2018	2191.7	150962
	2017	2194.4	136172
	2016	2195.4	123391
	2015	2188.3	113692
	2014	2171.1	106732
	2013	2125.4	100569
Bingzhou	2019	392.3	62639
Baoji	2018	377.1	60227
Chengde	2019	358.3	41080
Chengdu	2019	1500.1	103386
	2018	1476.1	96982
	2016	1398.9	75089
Changzhou	2016	470.8	122631
Guiyang	2019	497.2	81995
Hefei	2020	937.3	106745
	2019	770.4	121621
Hangzhou	2020	813.8	136617
Handan	2019	955.0	36546
Hohhot	2019	313.7	89138
Jinan	2021	933.6	123075
Jiaozuo	2021	352.0	60643
Lanzhou	2021	438.4	73807
Langfang	2019	492.1	65512
Lvliang	2019	389.1	38758
Linfen	2019	391.2	48438
Leshan	2017	351.8	46130
Nanjing	2021	942.3	173560
	2020	932.0	158620
	2019	850.0	165060
	2013	818.8	98685
Nanping	2017	319.0	60694
Rizhao	2022	296.8	77669
	2021	297.2	74434
Suzhou	2022	1291.1	185571
Shaoxing	2021	446.9	127875
Shijiazhuang	2020	1123.5	52961
	2019	1103.1	52859
Shanghai	2017	2418.3	126634
Tongchuan	2017	83.3	39339
Weihai	2021	291.5	118925
Wuhu	2021	367.2	117174
Wuhan	2020	1244.8	131441
	2017	1089.3	120880
Weinan	2017	538.3	30156
Xinxiang	2021	617.0	52028
	2020	626.0	46570

Xi'an	2019	1020.4	77494
	2017	961.7	66649
Xingtai	2019	739.5	28707
Xianyang	2018	436.6	52339
Yibin	2022	548.4	74341
Yantai	2021	708.3	122818
Yulin	2021	363.0	149899
Yuncheng	2019	537.3	30592
Zhengzhou	2021	1274.0	100092
	2019	1035.2	113139
	2018	1013.6	106611
Zhangjiakou	2019	442.3	35025

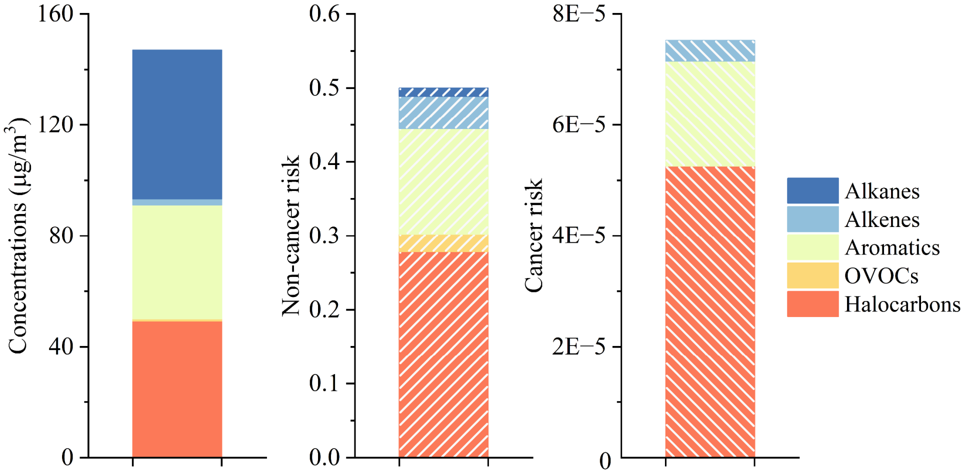

Figure S1. Concentrations (left), non-cancer risk (middle) and cancer risk (right) of different atmospheric hazardous VOC groups in China.

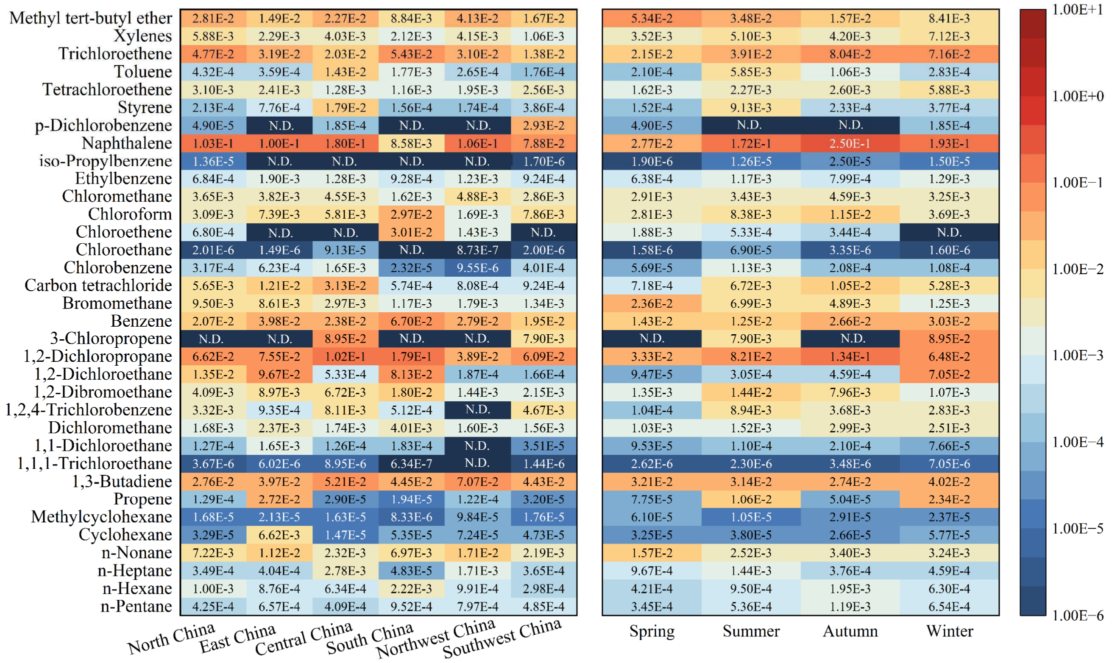

Figure S2. The non-cancer risk of different regions and seasons in China (N.D. represents not detected).

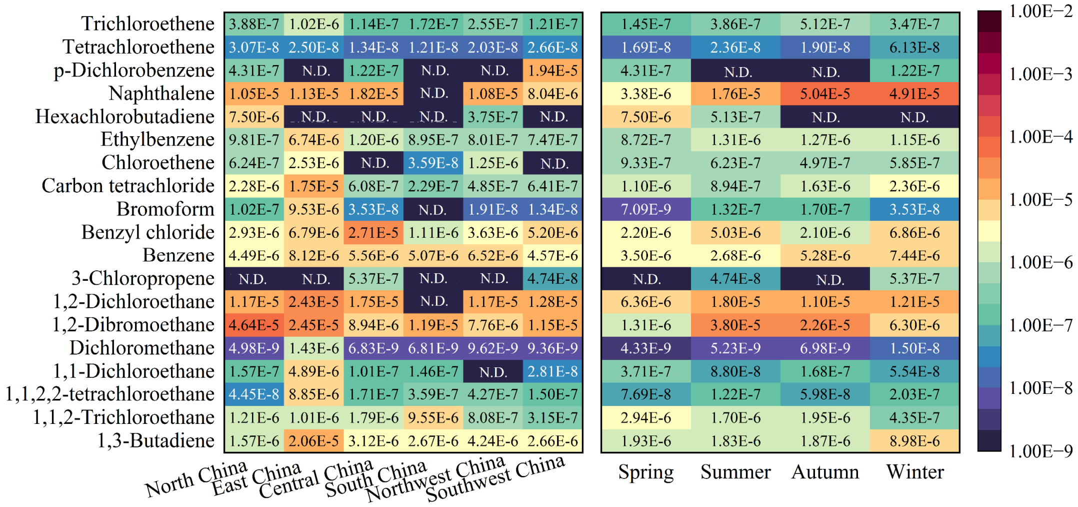

Figure S3. The cancer risk of different regions and seasons in China (N.D. represents not detected).

REFERENCES

1. Zhang, X.; Wang, J.; Zhao, J.; He, J.; Lei, Y.; Meng, K.; Wei, R.; Zhang, X.; Zhang, M.; Ni, S.; et al. Chemical Characteristics and Sources Apportionment of Volatile Organic Compounds in the Primary Urban Area of Shijiazhuang, North China Plain. *J Environ Sci* **2025**, *149*, 465–475, doi:10.1016/j.jes.2024.01.009.
2. Wang, X.; Han, Y.; Tu, X.; Shen, J.; Zhang, B.; Fu, H. Distribution of Volatile Organic Compounds (VOCs) in the Urban Atmosphere of Hangzhou, East China: Temporal Variation, Source Attribution, and Impact on the Secondary Formations of Ozone and Aerosol. *Front Env Sci-Switz* **2024**, *12*, 1418948, doi:10.3389/fenvs.2024.1418948.
3. Wang, B.; Sun, Y.; Sun, L.; Liu, Z.; Wang, C.; Zhang, R.; Zhu, C.; Yang, N.; Fan, G.; Sun, X.; et al. Causes of Summer Ozone Pollution Events in Jinan, East China: Local Photochemical Formation or Regional Transport? *Atmosphere-Basel* **2024**, *15*, 232, doi:10.3390/atmos15020232.
4. Hao, S.; Du, Q.; Wei, X.; Yan, H.; Zhang, M.; Sun, Y.; Liu, S.; Fan, L.; Zhang, G. Composition and Reactivity of Volatile Organic Compounds and the Implications for Ozone Formation in the North China Plain. *Atmosphere-Basel* **2024**, *15*, 213, doi:10.3390/atmos15020213.
5. Ren, H.; Xia, Z.; Yao, L.; Qin, G.; Zhang, Y.; Xu, H.; Wang, Z.; Cheng, J. Investigation on Ozone Formation Mechanism and Control Strategy of VOCs in Petrochemical Region: Insights from Chemical Reactivity and Photochemical Loss. *Sci Total Environ* **2024**, *914*, 169891, doi:10.1016/j.scitotenv.2024.169891.
6. Zhang, Z.; Zhang, Y.; Zhong, S.; Jie, F.; Bai, B.; Huang, C.; Ge, X. Anthropogenic-Driven Changes in Concentrations and Sources of Winter Volatile Organic Compounds in an Urban Environment in the Yangtze River Delta of China between 2013 and 2021. *Sci Total Environ* **2024**, *942*, 173713, doi:10.1016/j.scitotenv.2024.173713.
7. Lyu, Y.; Gao, Y.; Pang, X.; Sun, S.; Luo, P.; Cai, D.; Qin, K.; Wu, Z.; Wang, B. Elucidating Contributions of Volatile Organic Compounds to Ozone Formation Using Random Forest during COVID-19 Pandemic: A Case Study in China. *Environ Pollut* **2024**, *346*, 123532, doi:10.1016/j.envpol.2024.123532.
8. Yan, D.; Wang, Y.; Bai, Z.; Cheng, M.; Tang, G.; Liu, Y.; Zhuoga, D.; Yu, H.; Bian, J.; Wang, Y. Vertical Distribution of VOCs in the Boundary Layer of the Lhasa Valley and Its Impact on Ozone Pollution. *Environ Pollut* **2024**, *340*, 122786, doi:10.1016/j.envpol.2023.122786.
9. Zuo, H.; Jiang, Y.; Yuan, J.; Wang, Z.; Zhang, P.; Guo, C.; Wang, Z.; Chen, Y.; Wen, Q.; Wei, Y.; et al. Pollution Characteristics and Source Differences of VOCs before and after COVID-19 in Beijing. *Sci Total Environ* **2024**, *907*, 167694, doi:10.1016/j.scitotenv.2023.167694.
10. Liao, D.; Wang, L.; Wang, Y.; Lin, C.; Chen, J.; Huang, H.; Zhang, Z.; Choi, S.-D.; Hong, Y. Health Risks and Environmental Influence of Volatile Organic Compounds (VOCs) in a Residential Area near an Industrial Park in Southeast China. *Atmos Pollut Res* **2024**, *15*, 101966, doi:10.1016/j.apr.2023.101966.
11. Li, P.; Chen, C.; Liu, D.; Lian, J.; Li, W.; Fan, C.; Yan, L.; Gao, Y.; Wang, M.; Liu, H.; et al. Characteristics and Source Apportionment of Ambient Volatile Organic Compounds and Ozone Generation Sensitivity in Urban Jiaozuo, China. *J Environ Sci* **2024**, *138*, 607–625, doi:10.1016/j.jes.2023.04.016.
12. Liu, Y.; Yin, S.; Zhang, S.; Ma, W.; Zhang, X.; Qiu, P.; Li, C.; Wang, G.; Hou, D.; Zhang,

xiang; et al. Drivers and Impacts of Decreasing Concentrations of Atmospheric Volatile Organic Compounds (VOCs) in Beijing during 2016–2020. *Sci Total Environ* **2024**, *906*, 167847, doi:10.1016/j.scitotenv.2023.167847.

13. Xie, S.; Gong, Y.; Chen, Y.; Li, K.; Liu, J. Characterization and Source Analysis of Pollution Caused by Atmospheric Volatile Organic Compounds in the Spring, Kunming, China. *Atmosphere-Basel* **2023**, *14*, 1767, doi:10.3390/atmos14121767.
14. Liu, Z.; Zha, F.; Wang, Y.; Yuan, B.; Liu, B.; Tang, G. Vertical Evolution of the Concentrations and Sources of Volatile Organic Compounds in the Lower Boundary Layer in Urban Beijing in Summer. *Chemosphere* **2023**, *332*, 138767, doi:10.1016/j.chemosphere.2023.138767.
15. Yu, H.; Liu, Q.; Wei, N.; Hu, M.; Xu, X.; Wang, S.; Zhou, J.; Zhao, W.; Zhang, W. Investigation of Summertime Ozone Formation and Sources of Volatile Organic Compounds in the Suburb Area of Hefei: A Case Study of 2020. *Atmosphere-Basel* **2023**, *14*, 740, doi:10.3390/atmos14040740.
16. Luo, S.; Hao, Q.; Xu, Z.; Zhang, G.; Liang, Z.; Gou, Y.; Wang, X.; Chen, F.; He, Y.; Jiang, C. Composition Characteristics of VOCs in the Atmosphere of the Beibei Urban District of Chongqing: Insights from Long-Term Monitoring. *Atmosphere-Basel* **2023**, *14*, 1452, doi:10.3390/atmos14091452.
17. Wang, B.; Liu, Z.; Li, Z.; Sun, Y.; Wang, C.; Zhu, C.; Sun, L.; Yang, N.; Bai, G.; Fan, G.; et al. Characteristics, Chemical Transformation and Source Apportionment of Volatile Organic Compounds (VOCs) during Wintertime at a Suburban Site in a Provincial Capital City, East China. *Atmos Environ* **2023**, *298*, 119621, doi:10.1016/j.atmosenv.2023.119621.
18. Wang, W.; Fang, H.; Zhang, Y.; Ding, Y.; Hua, F.; Wu, T.; Yan, Y. Characterizing Sources and Ozone Formations of Summertime Volatile Organic Compounds Observed in a Medium-Sized City in Yangtze River Delta Region. *Chemosphere* **2023**, *328*, 138609, doi:10.1016/j.chemosphere.2023.138609.
19. Liu, Z.; Hu, K.; Zhang, K.; Zhu, S.; Wang, M.; Li, L. VOCs Sources and Roles in O3 Formation in the Central Yangtze River Delta Region of China. *Atmos Environ* **2023**, *302*, 119755, doi:10.1016/j.atmosenv.2023.119755.
20. Zhang, H.; Yin, S.; Xu, Y.; Zhang, D.; Yu, S.; Lu, X.; Xin, K. Multiple Source Apportionments, Secondary Transformation Potential and Human Exposure of VOCs: A Case Study in a Megacity of China. *Atmos Res* **2023**, *291*, 106823, doi:10.1016/j.atmosres.2023.106823.
21. Xu, K.; Liu, Y.; Li, C.; Zhang, C.; Liu, X.; Li, Q.; Xiong, M.; Zhang, Y.; Yin, S.; Ding, Y. Enhanced Secondary Organic Aerosol Formation during Dust Episodes by Photochemical Reactions in the Winter in Wuhan. *J Environ Sci* **2023**, *133*, 70–82, doi:10.1016/j.jes.2022.04.018.
22. Han J.; Liu Z.; Hu B.; Zhu W.; Tang G.; Liu Q.; Ji D.; Wang Y. Observations and explicit modeling of summer and autumn ozone formation in urban Beijing: Identification of key precursor species and sources. *Atmos Environ* **2023**, *309*, 119932, doi:10.1016/j.atmosenv.2023.119932.
23. Wang, D.; Zhou, J.; Han, L.; Tian, W.; Wang, C.; Li, Y.; Chen, J. Source Apportionment of VOCs and Ozone Formation Potential and Transport in Chengdu, China. *Atmos Pollut Res* **2023**, *14*, 101730, doi:10.1016/j.apr.2023.101730.

24. Zhang, X.; Ma, Q.; Chu, W.; Ning, M.; Liu, X.; Xiao, F.; Cai, N.; Wu, Z.; Yan, G. Identify the Key Emission Sources for Mitigating Ozone Pollution: A Case Study of Urban Area in the Yangtze River Delta Region, China. *Sci Total Environ* **2023**, *892*, 164703, doi:10.1016/j.scitotenv.2023.164703.
25. Guan, Y.; Liu, X.; Zheng, Z.; Dai, Y.; Du, G.; Han, J.; Hou, L.; Duan, E. Summer O3 Pollution Cycle Characteristics and VOCs Sources in a Central City of Beijing-Tianjin-Hebei Area, China. *Environ Pollut* **2023**, *323*, 121293, doi:10.1016/j.envpol.2023.121293.
26. Zheng, H.; Chen, D.; Qian, D.; Meng, K.; Hu, C.; Li, S.; Feng, X.; Xu, H.; Peng, Q.; Yu, H.; et al. Source Analysis and Control Strategy of VOCs and PM2.5 in High Ozone Season in Industrial Zone of Shaoxing City, China. *Water Air Soil Pollut* **2023**, *234*, 1–14, doi:10.1007/s11270-023-06324-6.
27. Zhang, Z.; Sun, Y.; Li, J. Characteristics and Sources of VOCs in a Coastal City in Eastern China and the Implications in Secondary Organic Aerosol and O3 Formation. *Sci Total Environ* **2023**, *887*, 164117, doi:10.1016/j.scitotenv.2023.164117.
28. Wang, B.; Li, Z.; Liu, Z.; Sun, Y.; Wang, C.; Xiao, Y.; Lu, X.; Yan, G.; Xu, C. Characteristics, Secondary Transformation Potential and Health Risks of Atmospheric Volatile Organic Compounds in an Industrial Area in Zibo, East China. *Atmosphere-Basel* **2023**, *14*, 158, doi:10.3390/atmos14010158.
29. Ye, C.; Guo, S.; Lin, W.; Tian, F.; Wang, J.; Zhang, C.; Chi, S.; Chen, Y.; Zhang, Y.; Zeng, L.; et al. Measurement Report: Source Apportionment and Environmental Impacts of Volatile Organic Compounds (VOCs) in Lhasa, a Highland City in China. *Atmos Chem Phys* **2023**, *23*, 10383–10397, doi:10.5194/acp-23-10383-2023.
30. Wang, Z.; Zhang, P.; Pan, L.; Qian, Y.; Li, Z.; Li, X.; Guo, C.; Zhu, X.; Xie, Y.; Wei, Y. Ambient Volatile Organic Compound Characterization, Source Apportionment, and Risk Assessment in Three Megacities of China in 2019. *Toxics* **2023**, *11*, 651, doi:10.3390/toxics11080651.
31. Zeng, X.; Han, M.; Ren, G.; Liu, G.; Wang, X.; Du, K.; Zhang, X.; Lin, H. A Comprehensive Investigation on Source Apportionment and Multi-Directional Regional Transport of Volatile Organic Compounds and Ozone in Urban Zhengzhou. *Chemosphere* **2023**, *334*, 139001, doi:10.1016/j.chemosphere.2023.139001.
32. Li, B.; Ho, S.S.H.; Li, X.; Guo, L.; Feng, R.; Fang, X. Pioneering Observation of Atmospheric Volatile Organic Compounds in Hangzhou in Eastern China and Implications for Upcoming 2022 Asian Games. *J Environ Sci* **2023**, *124*, 723–734, doi:10.1016/j.jes.2021.12.029.
33. Wu, Y.; Fan, X.; Liu, Y.; Zhang, J.; Wang, H.; Sun, L.; Fang, T.; Mao, H.; Hu, J.; Wu, L.; et al. Source Apportionment of VOCs Based on Photochemical Loss in Summer at a Suburban Site in Beijing. *Atmos Environ* **2023**, *293*, 119459, doi:10.1016/j.atmosenv.2022.119459.
34. Zhang, Y.; Li, F.; Cheng, Q.; Zhang, C.; Liu, Y.; Li, Q.; Yin, S.; Zhang, S.; Liu, X. Characteristics and Secondary Transformation Potential of Volatile Organic Compounds in Wuhan, China. *Atmos Environ* **2023**, *294*, 119469, doi:10.1016/j.atmosenv.2022.119469.
35. Xu, Z.; Zou, Q.; Jin, L.; Shen, Y.; Shen, J.; Xu, B.; Qu, F.; Zhang, F.; Xu, J.; Pei, X.; et al. Characteristics and Sources of Ambient Volatile Organic Compounds (VOCs) at a Regional Background Site, YRD Region, China: Significant Influence of Solvent Evaporation during Hot Months. *Sci Total Environ* **2023**, *857*, 159674, doi:10.1016/j.scitotenv.2022.159674.
36. Yang, Y.; Guo, W.; Sun, J.; Chen, Q.; Meng, X.; Wang, L.; Tao, H.; Yang, L. Characteristics of

Volatile Organic Compounds and Secondary Organic Aerosol Pollution in Different Functional Areas of Petrochemical Industrial Cities in Northwest China. *Sci Total Environ* **2023**, *858*, 159903, doi:10.1016/j.scitotenv.2022.159903.

37. Liu, C.; Xin, Y.; Zhang, C.; Liu, J.; Liu, P.; He, X.; Mu, Y. Ambient Volatile Organic Compounds in Urban and Industrial Regions in Beijing: Characteristics, Source Apportionment, Secondary Transformation and Health Risk Assessment. *Sci Total Environ* **2023**, *855*, 158873, doi:10.1016/j.scitotenv.2022.158873.
38. Kong, L.; Luo, T.; Jiang, X.; Zhou, S.; Huang, G.; Chen, D.; Lan, Y.; Yang, F. Seasonal Variation Characteristics of VOCs and Their Influences on Secondary Pollutants in Yibin, Southwest China. *Atmosphere-Basel* **2022**, *13*, 1389, doi:10.3390/atmos13091389.
39. Liu, Y.; Qiu, P.; Xu, K.; Li, C.; Yin, S.; Zhang, Y.; Ding, Y.; Zhang, C.; Wang, Z.; Zhai, R.; et al. Analysis of VOC Emissions and O3 Control Strategies in the Fenhe Plain Cities, China. *J Environ Manage* **2023**, *325*, 116534, doi:10.1016/j.jenvman.2022.116534.
40. Huang, H.; Wang, Z.; Guo, J.; Wang, C.; Zhang, X. Composition, Seasonal Variation and Sources Attribution of Volatile Organic Compounds in Urban Air in Southwestern China. *Urban Clim* **2022**, *45*, 101241, doi:10.1016/j.uclim.2022.101241.
41. Huang, A.; Yin, S.; Yuan, M.; Xu, Y.; Yu, S.; Zhang, D.; Lu, X.; Zhang, R. Characteristics, Source Analysis and Chemical Reactivity of Ambient VOCs in a Heavily Polluted City of Central China. *Atmos Pollut Res* **2022**, *13*, 101390, doi:10.1016/j.apr.2022.101390.
42. Xue, Y.; Wang, L.; Liu, S.; Huang, Y.; Chen, L.; Cui, L.; Cao, J. Upward Trend and Formation of Surface Ozone in the Guanzhong Basin, Northwest China. *J Hazard Mater* **2022**, *427*, 128175, doi:10.1016/j.jhazmat.2021.128175.
43. Yao, D.; Li, C.; Niu, Q.; Gao, W.; Yu, H.; Yan, G.; Liu, J.; Cao, Z.; Wang, S.; Wang, Y. Characteristics of Volatile Organic Compounds and Their Contribution to Secondary Organic Aerosols during the High O3 Period in a Central Industry City in China. *Atmosphere-Basel* **2022**, *13*, 1625, doi:10.3390/atmos13101625.
44. Liu, Y.; Qiu, P.; Li, C.; Li, X.; Ma, W.; Yin, S.; Yu, Q.; Li, J.; Liu, X. Evolution and Variations of Atmospheric VOCs and O3 Photochemistry during a Summer O3 Event in a County-Level City, Southern China. *Atmos Environ* **2022**, *272*, 118942, doi:10.1016/j.atmosenv.2022.118942.
45. Zhou, B.; Zhao, T.; Ma, J.; Zhang, Y.; Zhang, L.; Huo, P.; Zhang, Y. Characterization of VOCs during Nonheating and Heating Periods in the Typical Suburban Area of Beijing, China: Sources and Health Assessment. *Atmosphere-Basel* **2022**, *13*, 560, doi:10.3390/atmos13040560.
46. Chen, T.; Huang, L.; Zhang, X.; Gao, R.; Li, H.; Fan, K.; Ma, D.; Ma, Z.; Xue, L.; Wang, W. Effects of Coal Chemical Industry on Atmospheric Volatile Organic Compounds Emission and Ozone Formation in a Northwestern Chinese City. *Sci Total Environ* **2022**, *839*, 156149, doi:10.1016/j.scitotenv.2022.156149.
47. Li, Y.; Liu, Y.; Hou, M.; Huang, H.; Fan, L.; Ye, D. Characteristics and Sources of Volatile Organic Compounds (VOCs) in Xinxiang, China, during the 2021 Summer Ozone Pollution Control. *Sci Total Environ* **2022**, *842*, 156746, doi:10.1016/j.scitotenv.2022.156746.
48. Li, J.; Deng, S.; Tohti, A.; Li, G.; Yi, X.; Lu, Z.; Liu, J.; Zhang, S. Spatial Characteristics of VOCs and Their Ozone and Secondary Organic Aerosol Formation Potentials in Autumn and Winter in the Guanzhong Plain, China. *Environ Res* **2022**, *211*, 113036,

doi:10.1016/j.envres.2022.113036.

49. Guo, W.; Yang, Y.; Chen, Q.; Zhu, Y.; Zhang, Y.; Zhang, Y.; Liu, Y.; Li, G.; Sun, W.; She, J. Chemical Reactivity of Volatile Organic Compounds and Their Effects on Ozone Formation in a Petrochemical Industrial Area of Lanzhou, Western China. *Sci Total Environ* **2022**, *839*, 155901, doi:10.1016/j.scitotenv.2022.155901.
50. Li, C.; Liu, Y.; Cheng, B.; Zhang, Y.; Liu, X.; Qu, Y.; An, J.; Kong, L.; Zhang, Y.; Zhang, C.; et al. A Comprehensive Investigation on Volatile Organic Compounds (VOCs) in 2018 in Beijing, China: Characteristics, Sources and Behaviours in Response to O3 Formation. *Sci Total Environ* **2022**, *806*, 150247, doi:10.1016/j.scitotenv.2021.150247.
51. Cui, L.; Wu, D.; Wang, S.; Xu, Q.; Hu, R.; Hao, J. Measurement Report: Ambient Volatile Organic Compound (VOC) Pollution in Urban Beijing: Characteristics, Sources, and Implications for Pollution Control. *Atmos Chem Phys* **2022**, *22*, 11931–11944, doi:10.5194/acp-22-11931-2022.
52. Manager, J. Spatial-Temporal Characteristics and Source Apportionment of Ambient VOCs in Southeast Mountain Area of China. *Aerosol Air Qual Res* **2022**, *22*, 220016, doi:10.4209/aaqr.220016.
53. Li, J.; Deng, S.; Li, G.; Lu, Z.; Song, H.; Gao, J.; Sun, Z.; Xu, K. VOCs Characteristics and Their Ozone and SOA Formation Potentials in Autumn and Winter at Weinan, China. *Environ Res* **2022**, *203*, 111821, doi:10.1016/j.envres.2021.111821.
54. Wang, S.; Liu, G.; Zhang, H.; Yi, M.; Liu, Y.; Hong, X.; Bao, X. Insight into the Environmental Monitoring and Source Apportionment of Volatile Organic Compounds (VOCs) in Various Functional Areas. *Air Qual Atmos Health* **2022**, *15*, 1121–1131, doi:10.1007/s11869-021-01090-y.
55. Li, Y.; Gao, R.; Xue, L.; Wu, Z.; Yang, X.; Gao, J.; Ren, L.; Li, H.; Ren, Y.; Li, G.; et al. Ambient Volatile Organic Compounds at Wudang Mountain in Central China: Characteristics, Sources and Implications to Ozone Formation. *Atmos Res* **2021**, *250*, 105359, doi:10.1016/j.atmosres.2020.105359.
56. Yao, D.; Tang, G.; Wang, Y.; Yang, Y.; Wang, L.; Chen, T.; He, H.; Wang, Y. Significant Contribution of Spring Northwest Transport to Volatile Organic Compounds in Beijing. *J Environ Sci* **2021**, *104*, 169–181, doi:10.1016/j.jes.2020.11.023.
57. Wang, Z.; Wang, H.; Zhang, L.; Guo, J.; Li, Z.; Wu, K.; Zhu, G.; Hou, D.; Su, H.; Sun, Z.; et al. Characteristics of Volatile Organic Compounds (VOCs) Based on Multisite Observations in Hebei Province in the Warm Season in 2019. *Atmos Environ* **2021**, *256*, 118435, doi:10.1016/j.atmosenv.2021.118435.
58. Xiong, C.; Wang, N.; Zhou, L.; Yang, F.; Qiu, Y.; Chen, J.; Han, L.; Li, J. Component Characteristics and Source Apportionment of Volatile Organic Compounds during Summer and Winter in Downtown Chengdu, Southwest China. *Atmos Environ* **2021**, *258*, 118485, doi:10.1016/j.atmosenv.2021.118485.
59. Liu, Y.; Kong, L.; Liu, X.; Zhang, Y.; Li, C.; Zhang, Y.; Zhang, C.; Qu, Y.; An, J.; Ma, D.; et al. Characteristics, Secondary Transformation, and Health Risk Assessment of Ambient Volatile Organic Compounds (VOCs) in Urban Beijing, China. *Atmos Pollut Res* **2021**, *12*, 33–46, doi:10.1016/j.apr.2021.01.013.
60. Yao, S.; Wang, Q.; Zhang, J.; Zhang, R.; Gao, Y.; Zhang, H.; Li, J.; Zhou, Z. Ambient Volatile Organic Compounds in a Heavy Industrial City: Concentration, Ozone Formation Potential,

Sources, and Health Risk Assessment. *Atmos Pollut Res* **2021**, *12*, 101053, doi:10.1016/j.apr.2021.101053.
61. Xie, F.; Zhou, X.; Wang, H.; Gao, J.; Hao, F.; He, J.; Lv, C. Heating Events Drive the Seasonal Patterns of Volatile Organic Compounds in a Typical Semi-Arid City. *Sci Total Environ* **2021**, *788*, 147781, doi:10.1016/j.scitotenv.2021.147781.
62. Xie, G.; Chen, H.; Zhang, F.; Shang, X.; Zhan, B.; Zeng, L.; Mu, Y.; Mellouki, A.; Tang, X.; Chen, J. Compositions, Sources, and Potential Health Risks of Volatile Organic Compounds in the Heavily Polluted Rural North China Plain during the Heating Season. *Sci Total Environ* **2021**, *789*, 147956, doi:10.1016/j.scitotenv.2021.147956.
63. Wang, M.; Lu, S.; Shao, M.; Zeng, L.; Zheng, J.; Xie, F.; Lin, H.; Hu, K.; Lu, X. Impact of COVID-19 Lockdown on Ambient Levels and Sources of Volatile Organic Compounds (VOCs) in Nanjing, China. *Sci Total Environ* **2021**, *757*, 143823, doi:10.1016/j.scitotenv.2020.143823.
64. Mozaffar, A.; Zhang, Y.-L.; Lin, Y.-C.; Xie, F.; Fan, M.-Y.; Cao, F. Measurement Report: High Contributions of Halocarbon and Aromatic Compounds to Atmospheric Volatile Organic Compounds in an Industrial Area. *Atmos Chem Phys* **2021**, *21*, 18087–18099, doi:10.5194/acp-21-18087-2021.
65. Yang, S.; Li, X.; Song, M.; Liu, Y.; Yu, X.; Chen, S.; Lu, S.; Wang, W.; Yang, Y.; Zeng, L.; et al. Characteristics and Sources of Volatile Organic Compounds during Pollution Episodes and Clean Periods in the Beijing-Tianjin-Hebei Region. *Sci Total Environ* **2021**, *799*, 149491, doi:10.1016/j.scitotenv.2021.149491.
66. Song, M.; Li, X.; Yang, S.; Yu, X.; Zhou, S.; Yang, Y.; Chen, S.; Dong, H.; Liao, K.; Chen, Q.; et al. Spatiotemporal Variation, Sources, and Secondary Transformation Potential of Volatile Organic Compounds in Xi'an, China. *Atmos Chem Phys* **2021**, *21*, 4939–4958, doi:10.5194/acp-21-4939-2021.
67. Zhang, C.; Liu, X.; Zhang, Y.; Tan, Q.; Feng, M.; Qu, Y.; An, J.; Deng, Y.; Zhai, R.; Wang, Z.; et al. Characteristics, Source Apportionment and Chemical Conversions of VOCs Based on a Comprehensive Summer Observation Experiment in Beijing. *Atmos Pollut Res* **2021**, *12*, 230–241, doi:10.1016/j.apr.2020.12.010.
68. Zhang, D.; He, B.; Yuan, M.; Yu, S.; Yin, S.; Zhang, R. Characteristics, Sources and Health Risks Assessment of VOCs in Zhengzhou, China during Haze Pollution Season. *J Environ Sci* **2021**, *108*, 44–57, doi:10.1016/j.jes.2021.01.035.
69. Jia, H.; Gao, S.; Duan, Y.; Fu, Q.; Che, X.; Xu, H.; Wang, Z.; Cheng, J. Investigation of Health Risk Assessment and Odor Pollution of Volatile Organic Compounds from Industrial Activities in the Yangtze River Delta Region, China. *Ecotox Environ Safe* **2021**, *208*, 111474, doi:10.1016/j.ecoenv.2020.111474.
70. Mozaffar, A.; Zhang, Y.-L.; Fan, M.; Cao, F.; Lin, Y.-C. Characteristics of Summertime Ambient VOCs and Their Contributions to O3 and SOA Formation in a Suburban Area of Nanjing, China. *Atmos Res* **2020**, *240*, 104923, doi:10.1016/j.atmosres.2020.104923.
71. Wang, W.; Xiong, T.; Zhang, W.; Luo, B.; Wang, D.; Jiang, X.; Rao, Z.; Jiang, Y.; Liu, Y.; Cheng, H.; et al. Observation and Analysis of VOCs in Nine Prefecture-Level Cities of Sichuan Province, China. *Environ Monit Assess* **2020**, *192*, 1–14, doi:10.1007/s10661-020-08360-9.
72. Tan, Q.; Liu, H.; Xie, S.; Zhou, L.; Song, T.; Shi, G.; Jiang, W.; Yang, F.; Wei, F. Temporal

and Spatial Distribution Characteristics and Source Origins of Volatile Organic Compounds in a Megacity of Sichuan Basin, China. *Environ Res* **2020**, *185*, 109478, doi:10.1016/j.envres.2020.109478.

73. Guan, Y.; Wang, L.; Wang, S.; Zhang, Y.; Xiao, J.; Wang, X.; Duan, E.; Hou, L. Temporal Variations and Source Apportionment of Volatile Organic Compounds at an Urban Site in Shijiazhuang, China. *J Environ Sci* **2020**, *97*, 25–34, doi:10.1016/j.jes.2020.04.022.
74. Li, C.; Li, Q.; Tong, D.; Wang, Q.; Wu, M.; Sun, B.; Su, G.; Tan, L. Environmental Impact and Health Risk Assessment of Volatile Organic Compound Emissions during Different Seasons in Beijing. *J Environ Sci* **2020**, *93*, 1–12, doi:10.1016/j.jes.2019.11.006.
75. Hui, L.; Liu, X.; Tan, Q.; Feng, M.; An, J.; Qu, Y.; Zhang, Y.; Deng, Y.; Zhai, R.; Wang, Z. VOC Characteristics, Chemical Reactivity and Sources in Urban Wuhan, Central China. *Atmos Environ* **2020**, *224*, 117340, doi:10.1016/j.atmosenv.2020.117340.
76. Liu, Y.; Song, M.; Liu, X.; Zhang, Y.; Hui, L.; Kong, L.; Zhang, Y.; Zhang, C.; Qu, Y.; An, J.; et al. Characterization and Sources of Volatile Organic Compounds (VOCs) and Their Related Changes during Ozone Pollution Days in 2016 in Beijing, China. *Environ Pollut* **2020**, *257*, 113599, doi:10.1016/j.envpol.2019.113599.
77. Tsai, J.-H.; Lu, Y.-T.; Chung, I.-I.; Chiang, H.-L. Traffic-Related Airborne VOC Profiles Variation on Road Sites and Residential Area within a Microscale in Urban Area in Southern Taiwan. *Atmosphere-Basel* **2020**, *11*, 1015, doi:10.3390/atmos11091015.
78. Fu, S.; Guo, M.; Luo, J.; Han, D.; Chen, X.; Jia, H.; Jin, X.; Liao, H.; Wang, X.; Fan, L.; et al. Improving VOCs Control Strategies Based on Source Characteristics and Chemical Reactivity in a Typical Coastal City of South China through Measurement and Emission Inventory. *Sci Total Environ* **2020**, *744*, 140825, doi:10.1016/j.scitotenv.2020.140825.
79. Simayi, M.; Shi, Y.; Xi, Z.; Yu, X.; Liu, H.; Tan, Q.; Song, D.; Zeng, L.; Lu, S.; Xie, S. Understanding the Sources and Spatiotemporal Characteristics of VOCs in the Chengdu Plain, China, through Measurement and Emission Inventory. *Sci Total Environ* **2020**, *714*, 136692, doi:10.1016/j.scitotenv.2020.136692.
80. Liang, Y.; Liu, X.; Wu, F.; Guo, Y.; Fan, X.; Xiao, H. The Year-Round Variations of VOC Mixing Ratios and Their Sources in Kuytun City (Northwestern China), near Oilfields. *Atmos Pollut Res* **2020**, *11*, 1513–1523, doi:10.1016/j.apr.2020.05.022.
81. Zhang, F.; Shang, X.; Chen, H.; Xie, G.; Fu, Y.; Wu, D.; Sun, W.; Liu, P.; Zhang, C.; Mu, Y.; et al. Significant Impact of Coal Combustion on VOCs Emissions in Winter in a North China Rural Site. *Sci Total Environ* **2020**, *720*, 137617, doi:10.1016/j.scitotenv.2020.137617.
82. Li, Y.; Yin, S.; Yu, S.; Yuan, M.; Dong, Z.; Zhang, D.; Yang, L.; Zhang, R. Characteristics, Source Apportionment and Health Risks of Ambient VOCs during High Ozone Period at an Urban Site in Central Plain, China. *Chemosphere* **2020**, *250*, 126283, doi:10.1016/j.chemosphere.2020.126283.
83. Sun, J.; Shen, Z.; Zhang, Y.; Zhang, Z.; Zhang, Q.; Zhang, T.; Niu, X.; Huang, Y.; Cui, L.; Xu, H.; et al. Urban VOC Profiles, Possible Sources, and Its Role in Ozone Formation for a Summer Campaign over Xi'an, China. *Environ Sci Pollut R* **2019**, *26*, 27769–27782, doi:10.1007/s11356-019-05950-0.
84. Song, M.; Liu, X.; Zhang, Y.; Shao, M.; Lu, K.; Tan, Q.; Feng, M.; Qu, Y. Sources and Abatement Mechanisms of VOCs in Southern China. *Atmos Environ* **2019**, *201*, 28–40, doi:10.1016/j.atmosenv.2018.12.019.

85. Deng, Y.; Li, J.; Li, Y.; Wu, R.; Xie, S. Characteristics of Volatile Organic Compounds, NO2, and Effects on Ozone Formation at a Site with High Ozone Level in Chengdu. *J Environ Sci* **2019**, *75*, 334–345, doi:10.1016/j.jes.2018.05.004.
86. Liu, Y.; Wang, H.; Jing, S.; Gao, Y.; Peng, Y.; Lou, S.; Cheng, T.; Tao, S.; Li, L.; Li, Y.; et al. Characteristics and Sources of Volatile Organic Compounds (VOCs) in Shanghai during Summer: Implications of Regional Transport. *Atmos Environ* **2019**, *215*, 116902, doi:10.1016/j.atmosenv.2019.116902.
87. Yang, Y.; Liu, X.; Zheng, J.; Tan, Q.; Feng, M.; Qu, Y.; An, J.; Cheng, N. Characteristics of One-Year Observation of VOCs, NOx, and O3 at an Urban Site in Wuhan, China. *J Environ Sci* **2019**, *79*, 297–310, doi:10.1016/j.jes.2018.12.002.
88. Widiana, D.R.; Wang, Y.C.; You, S.J.; Wang, Y.F. Source Apportionment and Health Risk Assessment of Ambient Volatile Organic Compounds in Primary Schools in Northern Taiwan. *Int J Environ Sci Te* **2019**, *16*, 6175–6188, doi:10.1007/s13762-018-2157-1.
89. Ge, Y.; Li, Q.; Wei, D.; Gao, L.; Tan, L.; Su, G.; Liu, G.; Liu, W.; Li, C.; Wang, Q. Emission Characteristics of 99 NMVOCs in Different Seasonal Days and the Relationship with Air Quality Parameters in Beijing, China. *Ecotox Environ Safe* **2019**, *169*, 797–806, doi:10.1016/j.ecoenv.2018.11.091.
90. Yang, Y.; Ji, D.; Sun, J.; Wang, Y.; Yao, D.; Zhao, S.; Yu, X.; Zeng, L.; Zhang, R.; Zhang, H.; et al. Ambient Volatile Organic Compounds in a Suburban Site between Beijing and Tianjin: Concentration Levels, Source Apportionment and Health Risk Assessment. *Sci Total Environ* **2019**, *695*, 133889, doi:10.1016/j.scitotenv.2019.133889.
91. Han, D.; Gao, S.; Fu, Q.; Cheng, J.; Chen, X.; Xu, H.; Liang, S.; Zhou, Y.; Ma, Y. Do Volatile Organic Compounds (VOCs) Emitted from Petrochemical Industries Affect Regional PM2.5? *Atmos Res* **2018**, *209*, 123–130, doi:10.1016/j.atmosres.2018.04.002.
92. Gao, J.; Zhang, J.; Li, H.; Li, L.; Xu, L.; Zhang, Y.; Wang, Z.; Wang, X.; Zhang, W.; Chen, Y.; et al. Comparative Study of Volatile Organic Compounds in Ambient Air Using Observed Mixing Ratios and Initial Mixing Ratios Taking Chemical Loss into Account – A Case Study in a Typical Urban Area in Beijing. *Sci Total Environ* **2018**, *628–629*, 791–804, doi:10.1016/j.scitotenv.2018.01.175.
93. Hui, L.; Liu, X.; Tan, Q.; Feng, M.; An, J.; Qu, Y.; Zhang, Y.; Jiang, M. Characteristics, Source Apportionment and Contribution of VOCs to Ozone Formation in Wuhan, Central China. *Atmos Environ* **2018**, *192*, 55–71, doi:10.1016/j.atmosenv.2018.08.042.
94. Li, J.; Zhai, C.; Yu, J.; Liu, R.; Li, Y.; Zeng, L.; Xie, S. Spatiotemporal Variations of Ambient Volatile Organic Compounds and Their Sources in Chongqing, a Mountainous Megacity in China. *Sci Total Environ* **2018**, *627*, 1442–1452, doi:10.1016/j.scitotenv.2018.02.010.
95. Zhang, H.; Li, H.; Zhang, Q.; Zhang, Y.; Zhang, W.; Wang, X.; Bi, F.; Chai, F.; Gao, J.; Meng, L.; et al. Atmospheric Volatile Organic Compounds in a Typical Urban Area of Beijing: Pollution Characterization, Health Risk Assessment and Source Apportionment. *Atmosphere-Basel* **2017**, *8*, 61, doi:10.3390/atmos8030061.
96. Wu, R.; Li, J.; Hao, Y.; Li, Y.; Zeng, L.; Xie, S. Evolution Process and Sources of Ambient Volatile Organic Compounds during a Severe Haze Event in Beijing, China. *Sci Total Environ* **2016**, *560–561*, 62–72, doi:10.1016/j.scitotenv.2016.04.030.
97. Lyu, X.P.; Chen, N.; Guo, H.; Zhang, W.H.; Wang, N.; Wang, Y.; Liu, M. Ambient Volatile Organic Compounds and Their Effect on Ozone Production in Wuhan, Central China. *Sci*

Total Environ **2016**, *541*, 200–209, doi:10.1016/j.scitotenv.2015.09.093.

98. Li, J.; Wu, R.; Li, Y.; Hao, Y.; Xie, S.; Zeng, L. Effects of Rigorous Emission Controls on Reducing Ambient Volatile Organic Compounds in Beijing, China. *Sci Total Environ* **2016**, *557–558*, 531–541, doi:10.1016/j.scitotenv.2016.03.140.
99. Li, J.; Xie, S.D.; Zeng, L.M.; Li, L.Y.; Li, Y.Q.; Wu, R.R. Characterization of Ambient Volatile Organic Compounds and Their Sources in Beijing, before, during, and after Asia-Pacific Economic Cooperation China 2014. *Atmos Chem Phys* **2015**, *15*, 7945–7959, doi:10.5194/acp-15-7945-2015.
